# Long-term exposure to a hypomagnetic field attenuates adult hippocampal neurogenesis and cognition

**DOI:** 10.1038/s41467-021-21468-x

**Published:** 2021-02-19

**Authors:** Bingfang Zhang, Lei Wang, Aisheng Zhan, Min Wang, Lanxiang Tian, Weixiang Guo, Yongxin Pan

**Affiliations:** 1 Biogeomagnetism Group, Key Laboratory of Earth and Planetary Physics, Institute of Geology and Geophysics, Chinese Academy of Sciences, Beijing, China; 2 Innovation Academy for Earth Science, Chinese Academy of Sciences, Beijing, China; 3 University of Chinese Academy of Sciences, Beijing, China; 4 State Key Laboratory for Molecular and Developmental Biology, Institute of Genetics and Developmental Biology, Chinese Academy of Sciences, Beijing, China; 5 The Paleomagnetism and Geochronology Laboratory, Institute of Geology and Geophysics, Chinese Academy of Sciences, Beijing, China

**Keywords:** Neural stem cells, Geomagnetism

## Abstract

Adult hippocampal neurogenesis contributes to learning and memory, and is sensitive to a variety of environmental stimuli. Exposure to a hypomagnetic field (HMF) influences the cognitive processes of various animals, from insects to human beings. However, whether HMF exposure affect adult hippocampal neurogenesis and hippocampus-dependent cognitions is still an enigma. Here, we showed that male C57BL/6 J mice exposed to HMF by means of near elimination of the geomagnetic field (GMF) exhibit significant impairments of adult hippocampal neurogenesis and hippocampus-dependent learning, which is strongly correlated with a reduction in the content of reactive oxygen species (ROS). However, these deficits seen in HMF-exposed mice could be rescued either by elevating ROS levels through pharmacological inhibition of ROS removal or by returning them back to GMF. Therefore, our results suggest that GMF plays an important role in adult hippocampal neurogenesis through maintaining appropriate endogenous ROS levels.

## Abstract (web summary)

Exposure to a hypomagnetic field (HMF) influences the cognitive processes of various animals, from insects to human beings. The authors show that in mice exposed to HMF, adult hippocampal neurogenesis and hippocampusdependent learning are impaired and could be rescued by restoring ROS levels.

## Introduction

On Earth, organisms are exposed to the geomagnetic field (GMF, present-day intensity value 25–65 μT), and many of them use the field for orientation and long-distance navigation^1^. Major effects of GMF on organisms include protecting them from solar wind and other cosmic radiations and preventing oxygen escape into interplanetary space, which helps to make the earth hospitable for living organisms^2,3^. However, organisms including human beings can be exposed to a hypomagnetic field (HMF, static magnetic field with an intensity <5 μT), e.g., in some artificial environments such as magnetically shielded rooms^4^ and during long-term deep space flights^5^. The HMF exposure may trigger central nervous system (CNS) dysfunction-like behaviors^6^, e.g., nervousness and stress-induced analgesia reduction in adult mice^7,8^, amnesia in chickens and Drosophila flies^9,10^, and impairments of cognitive processes in humans^11,12^. Although these studies provided great insights into the HMF effects on the CNS, the majority of them focused on behavioral analyses; thus, the underlying mechanisms are still elusive.

Adult hippocampus continuously generates new neurons throughout life, which are functionally integrated into hippocampal circuits and contribute to memory and learning^13,14^. The process of adult neurogenesis has been shown to be strongly influenced by physiological conditions and environmental interventions. For instance, learning^15,16^, exercise^17^, environmental enrichment^18^, and antidepressant treatments^19^, can increase hippocampal neurogenesis, while aging and stress show negative regulation on the proliferation of adult neural stem/progenitor cells (aNSCs) and survival of newborn neuron^17,20^. Therefore, identification of environmental factors that influence adult neurogenesis in the hippocampus may provide valuable insights into the functional integrity of the adult brain.

In this study, we sought to determine whether and how HMF exposure affects adult hippocampal neurogenesis and cognition. We found that long-term exposure to HMF impaired neurogenesis through decreasing aNSCs proliferation, altering cell lineages in critical development stages of neurogenesis, and impeding dendritic development of newborn neurons in the adult hippocampus, thereby resulted into defective cognition. Mechanistically, transcriptome analysis in combination with endogenous ROS in situ labeling using hydroethidine revealed reduced endogenous ROS levels in aNSCs of HMF-exposed mice. Furthermore, restoring ROS levels by pharmacological intervention in HMF-exposed mice rescued the defective adult hippocampal neurogenesis and cognition caused by long-term HMF exposure. Moreover, the defective adult hippocampal neurogenesis and cognition in HMF-exposed mice were able to be ameliorated by returning them back to GMF, which is accompanied by elevated ROS levels, but such beneficial effects could be blocked by reducing ROS levels.

## Results

### Long-term exposure to HMF impairs adult hippocampal neurogenesis

**Fig. 1:**
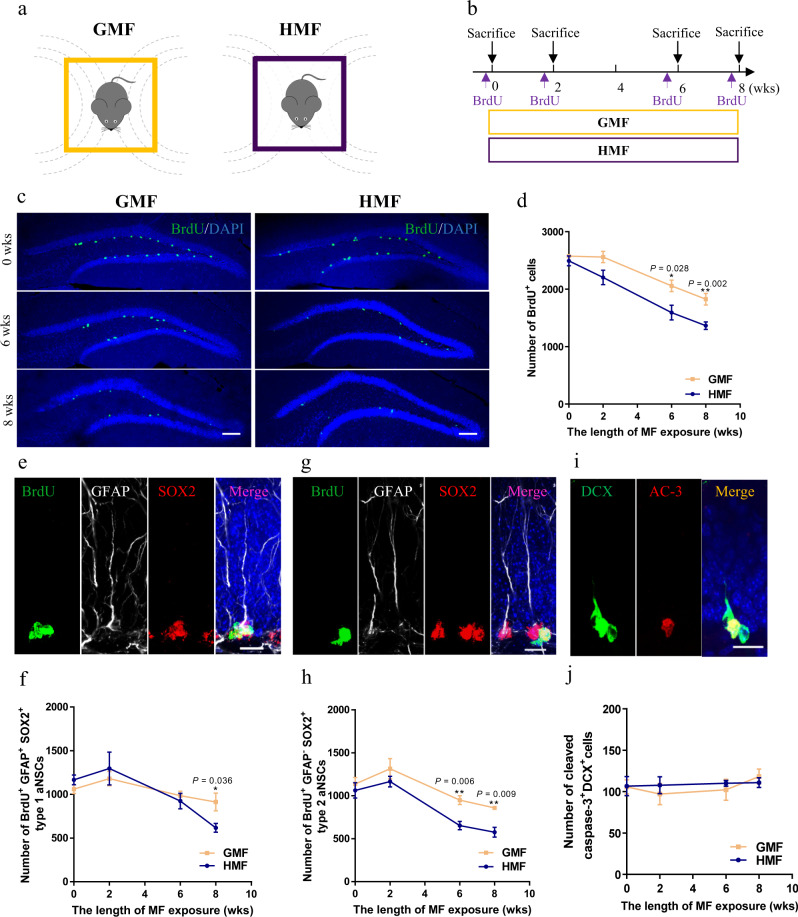
Long-term HMF exposure decreased the proliferation of aNSCs in the DG of mice. **a** Schematic diagram of magnetic field exposure to adult mice. **b** Experiments timeline for cell proliferation analysis during HMF or GMF exposure. **c** Representative images of BrdU^+^ cells in the DG after 2-h BrdU pulse labeling at 0-, 6-, 8-weeks of GMF- or HMF-exposure. Scale bar = 200 μm. **d** Quantification of numbers of BrdU^+^ cells in GMF- and HMF-exposed mice (GMF versus HMF, two-way ANOVA, F(1, 23) = 24.96, *P* < 0.0001). **e** Representative images of BrdU^+^GFAP^+^Sox2^+^ type 1 aNSCs in the adult DG. Scale bar = 20 μm. **f** Quantification of numbers of BrdU^+^GFAP^+^Sox2^+^ type 1 aNSCs in GMF- and HMF-exposed mice (GMF versus HMF, two-way ANOVA, F(1, 20) = 0.2978, *P* = 0.5913). **g** Representative images of BrdU^+^GFAP^-^Sox2^+^ type 2 aNSCs in the adult DG. Scale bar = 20 μm. **h** Quantification of numbers of BrdU^+^GFAP^−^Sox2^+^ type 2 aNSCs in GMF- and HMF-exposed mice (GMF versus HMF, two-way ANOVA, F(1, 21) = 14.81, *P* = 0.0009). **i** Representative images of brain section stained with DCX and activated caspase-3 (AC-3) in the adult DG. Scale bar = 20 μm. **j** Quantification of numbers of AC-3^+^DCX^+^ cells in GMF- and HMF-exposed mice (GMF versus HMF, two-way ANOVA, F(1, 24) = 0.186, *P* = 0.67). GMF, *n* = 4 mice, HMF, *n* = 4 mice. Data are presented as mean ± SEM. All data were analyzed by two-way ANOVA, and the two-tailed unpaired t test for two-group comparisons at each time point.

To determine whether HMF exposure affects adult hippocampal neurogenesis, we subjected adult mice to an HMF environment in custom-built tri-axis square Helmholtz coils^21^ (Fig. 1a and Supplementary Fig. 1a), in which the average magnetic strength of HMF (0.29 ± 0.01 μT) was ~190 times lower than the local GMF (55.26 ± 0.05 μT) (Supplementary Fig. 1b). However, the environmental electromagnetic field at different frequencies (Supplementary Fig. 1c), noise levels at different frequencies (Supplementary Fig. 1d), and light intensity (Supplementary Fig. 1e) were comparable in both GMF and HMF environments. Next, we tested the effect of exposure to HMF on aNSC proliferation in the dentate gyrus (DG) of adult mice. The mice were injected with bromodeoxyuridine (BrdU) and sacrificed 2 h later to examine aNSC proliferation during exposure to GMF or HMF (Fig. 1b). Compared with GMF groups, there was a decrease in the numbers of BrdU^+^ labeled proliferating cells in the DG of HMF-exposed mice at 2 weeks of HMF exposure and beyond (Fig. 1c, d). In addition, by analyzing the cell proliferation marker Ki67 in the DG, we found that the numbers of Ki67^+^ cells were also significantly reduced at 4 weeks of HMF exposure and beyond (Supplementary Fig. 2). During adult hippocampal neurogenesis, quiescent type 1 aNSCs generate proliferating type 2 aNSCs with transient amplifying characteristics, which give rise to neuroblasts that subsequently differentiate into mature neurons^14^. We then assessed the phenotypes of the BrdU labeled proliferating cells in the DG of GMF- and HMF-exposed mice. Compared to GMF-exposed controls, we found that there is a significant reduction in the numbers of BrdU^+^GFAP^+^Sox2^+^ type 1 aNSCs in HMF-exposed mice at 8-weeks HMF exposure (Fig. 1e, f), while such reduction effect on the numbers of BrdU^+^GFAP^-^Sox2^+^ type 2 aNSCs began at as early as 2-weeks exposure to HMF (Fig. 1g, h), suggesting that HMF exposure primarily affects proliferating type 2 aNSCs. However, no elevation caspase-3 activation was observed in DCX^+^ cells in the DG of HMF-exposed mice compared to GMF-exposed controls (Fig. 1i, j), which excluded the involvement of apoptosis on reduction of neurogenesis. Therefore, these data suggested that long-term exposure to HMF inhibits aNSC proliferation in adult DG.

**Fig. 2:**
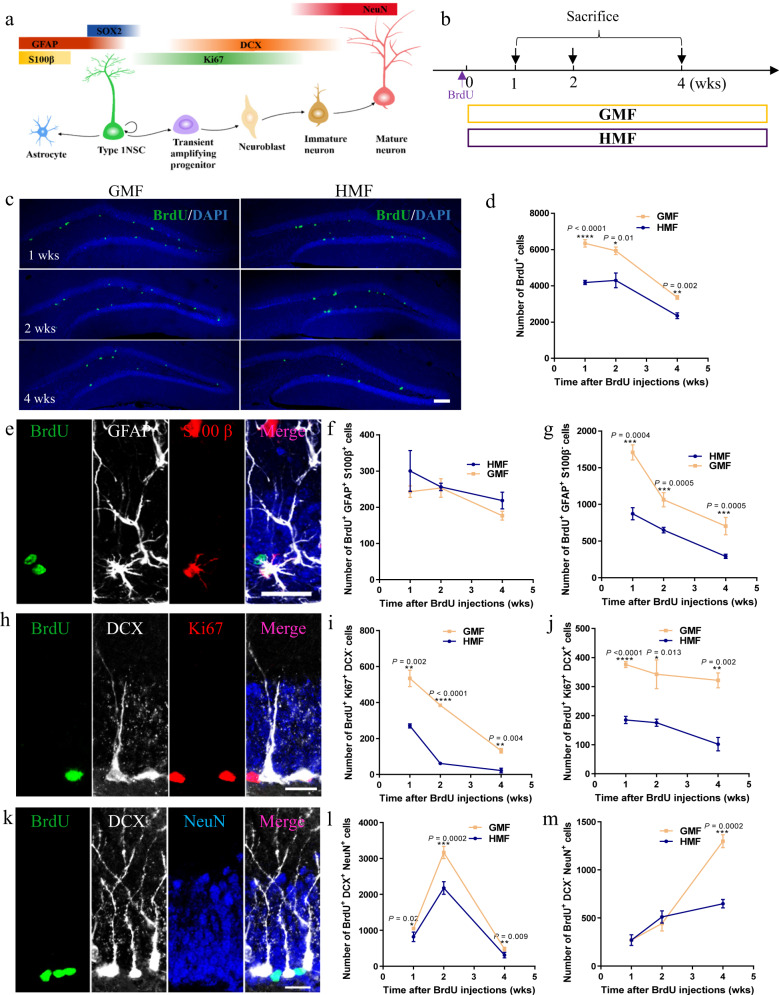
Long-term exposure to HMF impeded newborn neuronal differentiation in the DG of mice. **a** Schematic diagram showing the cell lineage-specific mapping markers across neurogenesis stages. **b** The experiment’s timeline for analyzing cell fate specification of aNSCs during HMF or GMF exposure. **c** Representative images of BrdU^+^ cells in the adult DG 1-, 2-, 4-weeks after BrdU injections. Scale bar = 200 μm. **d** Quantification of numbers of BrdU^+^ cells in GMF- and HMF-exposed mice 1-, 2-, 4-weeks after BrdU injections (GMF versus HMF, two-way ANOVA, F(1, 18) = 76.82, *P* < 0.0001). **e** Representative images of brain section stained with BrdU, GFAP, and S100β in the adult DG. Scale bar = 20 μm. **f**, **g** Quantification of numbers of BrdU^+^GFAP^+^S100β^+^ cells (**f**, GMF versus HMF, two-way ANOVA, F(1, 18) = 2.596, *P* = 0.1279) and BrdU^+^GFAP^+^S100β^–^ cells (**g**, GMF versus HMF, two-way ANOVA, F(1, 18) = 230.9, *P* < 0.0001) in GMF- and HMF-exposed mice 1-, 2-, 4-weeks after BrdU injections. **h** Representative images of brain section stained with BrdU, Ki67, and DCX in the adult DG. Scale bar = 20 μm. **i**, **j** Quantification of numbers of BrdU^+^Ki67^+^DCX^–^ cells (**i**, GMF versus HMF, two-way ANOVA, F(1, 18) = 88.54, *P* < 0.0001) and BrdU^+^Ki67^+^DCX^+^ (**j**, GMF versus HMF, two-way ANOVA, F(1, 18) = 88.54, *P* < 0.0001) cells in GMF- and HMF-exposed mice 1-, 2-, 4-weeks after BrdU injections. **k** Representative images of brain section stained with BrdU, DCX, and NeuN in the adult DG. Scale bar = 20 μm. **l**, **m** Quantification of numbers of BrdU^+^DCX^+^NeuN^+^ cells (**l**, GMF versus HMF, two-way ANOVA, F(1, 18) = 88.54, *P* < 0.0001) and BrdU^+^DCX^–^NeuN^+^ cells (**m**, GMF versus HMF, two-way ANOVA, F(1, 18) = 16.93, *P* = 0.0007) in GMF- and HMF-exposed mice 1-, 2-, 4-weeks after BrdU injections. GMF, *n* = 4 mice, HMF, *n* = 4 mice. Data are presented as mean ± SEM. All data were analyzed by two-way ANOVA, and the two-tailed unpaired *t* test for two-group comparisons at each time point.

To assess whether exposure to HMF affects the differentiation of aNSCs, we examined the cell lineages in critical developmental stages of hippocampal neurogenesis (Fig. 2a). Adult mice were pre-exposed to HMF for 8 weeks and sacrificed at 1, 2, and 4 weeks after BrdU injections during HMF exposure (Fig. 2b). The fate mapping of BrdU^+^ cells in the subgranular zone of the DG was examined. In general, HMF-exposed mice had significantly fewer BrdU^+^ cells 1-week post BrdU injections and beyond, compared to GMF-exposed controls (Fig. 2c, d). Starting from 1-week post BrdU injections and beyond, the numbers of BrdU^+^GFAP^+^S100β^−^ type 1 aNSCs (Fig. 2e, g), BrdU^+^Ki67^+^DCX^−^ transient amplifying cells (Fig. 2h, i), and BrdU^+^Ki67^+^DCX^+^ neuroblasts (Fig. 2h, j) were significantly lower in HMF-exposed mice than in GMF-exposed controls. Subsequently, the numbers of BrdU^+^DCX^+^NeuN^+^ post mitotic immature neurons (Fig. 2k, l) and BrdU^+^DCX^-^NeuN^+^ mature neurons (Fig. 2k, m) were also reduced in HMF-exposed mice 2-weeks and 4-weeks post BrdU injections, respectively. Moreover, the changes in the percentage of each neuronal lineage among the BrdU^+^ cells were closely similar to the changes in the number of each cell lineage (Supplementary Fig. 3a–e). Although there were no differences in the numbers of BrdU^+^GFAP^+^S100β^+^ astrocytes between the GMF and HMF groups (Fig. 2e, f), the percentage of BrdU^+^GFAP^+^S100β^+^ cells among BrdU^+^ cells was significantly higher in HMF-exposed mice (Supplementary Fig. 3f), suggesting that exposure to HMF most likely alters the fate specification of aNSCs. Nevertheless, the body weight (Supplementary Fig. 4a) and the DG volume (Supplementary Fig. 4b) were comparable between GMF- and HMF-exposed mice. Hence, these data suggest that long-term HMF exposure decreases aNSC proliferation and negatively alters the numbers of cell lineages in critical development stages of hippocampal neurogenesis, resulting in decreased neuronal differentiation.

### Long-term exposure to HMF impairs dendritic development of newborn neurons

**Fig. 3:**
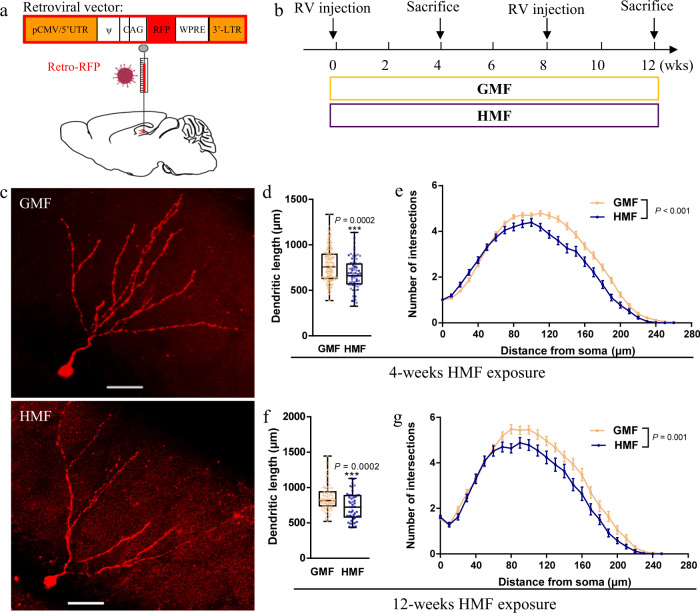
Long-term HMF exposure impaired the dendritic development of newborn neurons in the DG. **a** Schematic diagram of the retroviral vector and stereotaxic grafting of retrovirus into the DG of adult mice. **b** Experiments timeline for the in vivo labeling and analyses of newborn neurons in the DG of GMF- and HMF-exposed mice. **c** Representative images of RFP^+^ newborn neurons in the DG of GMF- and HMF-exposed mice at 4-weeks post retrovirus injection. Scale bar = 50 μm. **d**, **e** Quantification of the dendritic length (**d**) and dendritic complexity (**e**, GMF versus HMF, UNIANOVA, F(1, 242) = 15.19 *P* < 0.001) of RFP^+^ newborn neurons in the adult DG of mice during 4-weeks GMF and HMF exposure. GMF, *n* = 167 neurons from four mice, HMF, *n* = 77 neurons from four mice. Data were analyzed by UNIANOVA analysis, and the two-tailed unpaired *t* test for two-group comparisons. **f**, **g** Quantification of the dendritic length (**f**) and dendritic complexity (**g**, GMF versus HMF, UNIANOVA, F(1, 131) = 10.76, *P* = 0.001) of RFP^+^ newborn neurons in the adult DG of mice during 12-weeks GMF and HMF exposure. GMF, *n* = 82 neurons from four mice, HMF, *n* = 50 neurons from four mice. Data were analyzed by UNIANOVA analysis, and the two-tailed unpaired *t* test for two-group comparisons. The data in (**d**, **f**) were presented in the whisker plot with defined elements, median (centerline), upper and lower quartiles (bounds of box), and highest and lowest values (whiskers). Other data are presented as mean ± SEM.

Newborn neurons in the DG extend axonal and dendritic projections and establish new connections with the existing hippocampal circuitry to influence hippocampus-related functions^22^. We then examined the effect of exposure to HMF on the dendritic development of newborn neurons. Retroviruses expressing red fluorescent protein (RFP) were stereotactically injected into the DG of adult mice to label proliferating aNSCs and their progeny (Fig. 3a), and the RFP^+^ newborn neurons were evaluated at 4-weeks post retrovirus injection during HMF exposure (Fig. 3b). Compared to GMF-exposed mice, RFP^+^ neurons in mice during 4-weeks exposure to HMF displayed significant reductions of dendritic length and complexity at 4-weeks post retroviruses injection (Fig. 3c–e). To assess the long-term effect of exposure to HMF on the dendritic development of newborn neurons, retroviruses expressing RFP were injected into the DG of adult mice after 8-weeks of HMF exposure. In contrast to GMF-exposed controls, the dendritic length and complexity of the RFP^+^ newborn neurons in mice during 12-week exposure to HMF were also significantly decreased at 4-weeks post retroviruses injection (Fig. 3f, g). Thus, these data suggested that long-term exposure to HMF impairs the maturation of newborn neurons in adult DG.

### Long-term exposure to HMF decreases the cellular ROS levels in aNSC

**Fig. 4:**
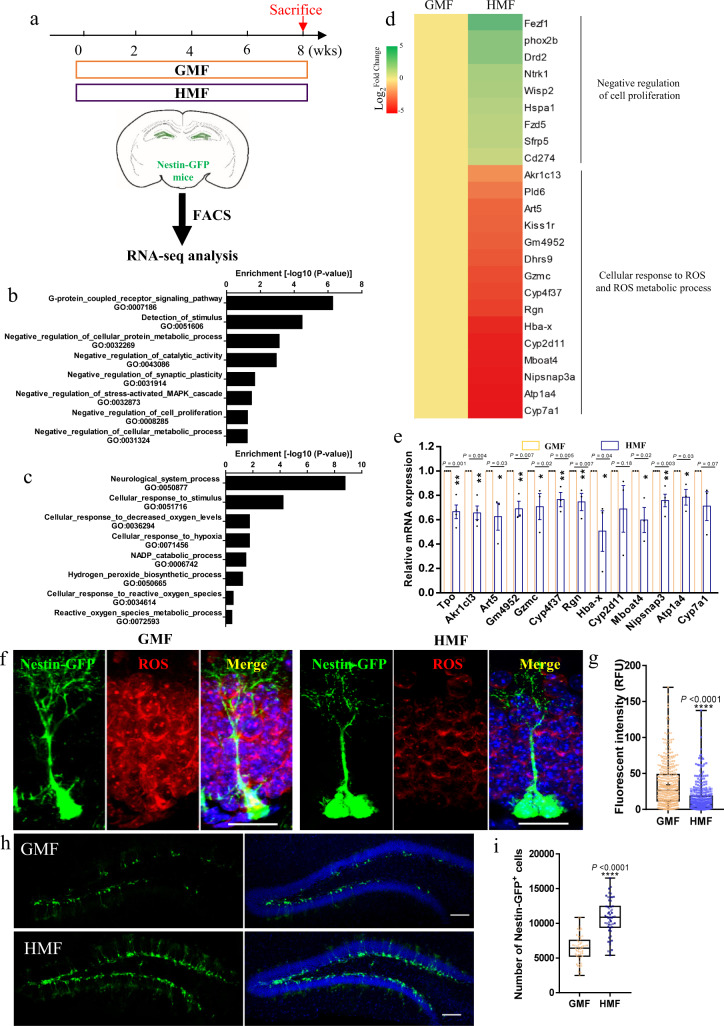
HMF exposure decreased ROS levels of adult NSCs. **a** Experimental timeline and strategy for isolation of aNSCs from the adult DG of Nestin-GFP mice using FACS, followed by RNA-sequencing. **b**, **c** Gene Ontology (GO) analysis of differentially expressed genes. Diagram of GO (Biological Process) terms that are significantly enriched in upregulated genes (**b**) or downregulated genes (**c**). **d** Difference in mRNA expression of ROS-related genes from aNSCs of GMF- and HMF-exposed mice. **e** RT-qPCR analysis of expression levels of selected genes associated with ROS synthesis and metabolism. Y-axis data is the relative mRNA expression ratio of HMF to GMF, and data of GMF is standardized to 1. *P* values were determined using two-tailed unpaired *t* test. **f** Hydroethidine fluorescence staining in the DG of Nestin-GFP mice after 8-weeks GMF and HMF exposure. Scale bar = 20 μm. **g** Quantification of hydroethidine fluorescence insensitive of GFP^+^ aNSCs in the DG of Nestin-GFP mice after 8-weeks GMF and HMF exposure, GMF, *n* = 400 cells from four mice, HMF, *n* = 400 cells from four mice. *P* values were determined using two-tailed unpaired *t* test. **h** Representative images of GFP^+^ cells in the DG of Nestin-GFP mice after 8-weeks GMF and HMF exposure. Scale bar = 100 μm. **i** Quantification of numbers of GFP^+^ cells of in the DG of Nestin-GFP mice after 8-weeks GMF and HMF exposure. GMF, *n* = 10 mice, HMF, *n* = 10 mice. *P* values were determined using two-tailed unpaired *t* test. The data in (**g**, **i**) were presented in the whisker plot with defined elements, median (centerline), upper and lower quartiles (bounds of box), and highest and lowest values (whiskers). Other data are presented as mean ± SEM.

To decipher the molecular mechanism underlying the influence of HMF exposure on adult hippocampal neurogenesis, we subjected adult transgenic mice expressing green fluorescent protein (GFP) under the control of the Nestin promoter (Nestin-GFP mice) to HMF or GMF, and subsequently isolated GFP^+^ aNSCs from the DG by fluorescence-activated cell sorting (Fig. 4a and Supplementary Fig. 13). RNA sequencing was performed to globally profile the transcriptomes of ten biological replicates per treatment (Supplementary Fig. 5a, b). Gene ontology analysis of the upregulated genes in aNSCs of HMF-exposed mice showed enrichment for gene signatures related to negative regulation of cellular protein metabolic processes and cell proliferation (Fig. 4b), while the downregulated genes were related to cellular response to hypoxia, cellular response to ROS, and ROS metabolic processes (Fig. 4c). The differential gene expression analysis revealed that aNSCs isolated from HMF-exposed mice expressed lower levels of ROS synthesis and metabolism-related genes compared to those from GMF-exposed mice (Fig. 4d), which were further validated by real-time quantitative polymerase chain reaction (RT-qPCR) analysis (Fig. 4e).

Previous studies suggested that weak magnetic fields can change endogenous ROS concentrations^23^, and proliferative, self-renewing NSCs need to maintain high ROS levels^24^. Based on the RNA analysis of the transcriptome, we hypothesized that HMF exposure decreases the cellular ROS content of the aNSCs in the DG. To directly measure the cellular ROS content in aNSCs in vivo, we performed in situ ROS labeling by injecting ROS-sensitive dye hydroethidine into adult mice. As expected, endogenous ROS levels were significantly decreased in the DG, as well as in GFP^+^ aNSCs of HMF-exposed Nestin-GFP mice compared to GMF-exposed controls (Fig. 4f, g). The cellular ROS in organisms is mainly removed by superoxide dismutases (SOD1, 2, 3) to maintain cellular oxidative homeostasis^25^. We then assessed whether HMF exposure affects the expression levels and activity of SODs in aNSCs by culturing them in a permalloy magnetic shielding chamber of a CO_2_ incubator. However, both GMF and HMF did not affect the relative mRNA expression levels of SODs by RT-qPCR assay (Supplementary Fig. 5c), as well as the protein expression levels by western blot assay (Supplementary Fig. 5d, e and Supplementary Fig.14) and ELISA assay (Supplementary Fig. 5f–h). Furthermore, the activities of CuZn-SOD (SOD1 and 3) and Mn-SOD (SOD2) were comparable between GFM- and HMF-exposed aNSCs (Supplementary Fig. 5i, j), which suggested that reducing ROS contents in aNSCs by HMF exposure is most likely due to decreasing ROS production rather than scavenging by SODs.

By taking advantage of Nestin-GFP mice to visualize aNSC with GFP fluorescence, we then assessed the effect of HMF exposure on the population of Nestin-GFP^+^ aNSCs in the DG. HMF-exposed Nestin-GFP mice had obviously higher numbers of GFP^+^ cells than GMF-exposed controls (Fig. 4h, i). During adult hippocampal neurogenesis, once quiescent type 1 aNSCs are activated, they then generate proliferating aNSCs with transient amplifying characteristics by consuming the aNSC pool^14^. By co-stained Ki67 with GFP, we found that the number of Ki67^+^ aNSCs were decreased in the HMF-exposed Nestin-GFP mice compared to the GMF controls (Supplementary Fig. 6b). Furthermore, we found that the Ki67^+^ aNSCs had higher ROS levels than Ki67^–^ radial glia-like cells (RGLs) in the HMF-exposed Nestin-GFP mice. Although Ki67^+^ aNSCs had higher ROS levels than Ki67^–^ aNSCs in HMF-exposed Nestin-GFP mice, their ROS levels in these two cell populations were relatively lower than their counterparts in GFM-exposed Nestin-GFP mice (Supplementary Fig. 6a, c). Therefore, these data therefore suggested that long-term exposure to HMF decreases aNSC proliferation most like through suppressing the activation of quiescent type 1 aNSCs.

### Pharmacological inhibition of ROS removal rescues defective adult hippocampal neurogenesis in HMF-exposed mice

**Fig. 5:**
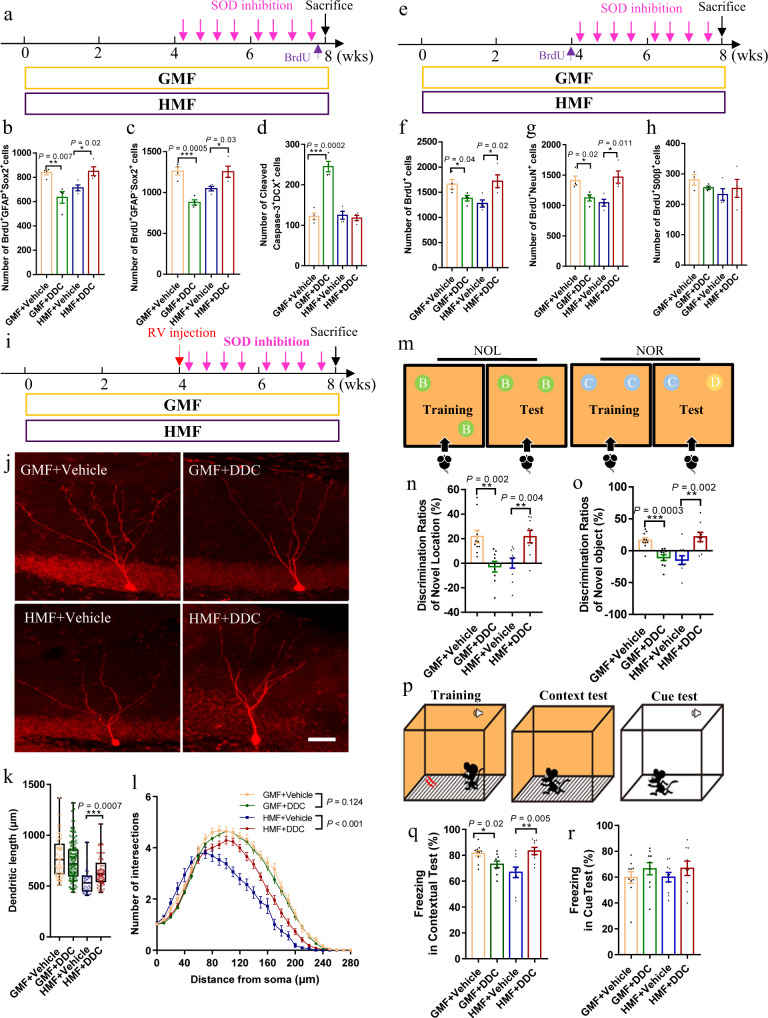
DDC treatment rescued the deficits of adult hippocampal neurogenesis, newborn neuron development, and hippocampus-dependent learning in HMF-exposed mice. **a** Experiments timeline for cell proliferation analysis in GFM- and HMF-exposed mice with or without DDC treatment. **b**–**d** Quantification of numbers of BrdU^+^Sox2^+^GFAP^+^ cells (**b**), BrdU^+^Sox2^+^GFAP^−^ cells (**c**), and active caspase-3^+^ DCX^+^ cells (**d**) in GFM- and HMF-exposed mice with or without DDC treatment. GMF + Vehicle, *n* = 4 mice, GMF + DDC, *n* = 4 mice, HMF + Vehicle, *n* = 4 mice, HMF + DDC, *n* = 4 mice. *P* values were determined using two-tailed unpaired *t* test. **e** Experiments timeline for cell differentiation analysis in GFM- and HMF-exposed mice with or without DDC treatment. **f**–**h** Quantification of numbers of BrdU^+^ cells (**f**), BrdU+NeuN^+^ cells (**g**), and BrdU^+^S100β^+^ (**h**) cells in GFM- and HMF-exposed mice with or without DDC treatment. GMF + Vehicle, *n* = 4 mice, GMF + DDC, *n* = 4 mice, HMF + Vehicle, *n* = 4 mice, HMF + DDC, *n* = 4 mice. *P* values were determined using two-tailed unpaired *t* test. **i** Timeline of experiments for the in vivo labeling and analyses of newborn neurons in the DG of mice, followed by DDC treatment. **j** Representative images of RFP^+^ newborn neurons in GFM- and HMF-exposed mice with or without DDC treatment. Scale bar = 50 μm. **k**, **l** Quantification of the dendritic length (**k**) and dendritic complexity (**l**, GMF + Vehicle versus GMF + DDC, UNIANOVA, F(1, 200) = 2.381 *p* = 0.124; HMF + Vehicle versus HMF + DDC, UNIANOVA, F(1, 113) = 23.84 *P* < 0.001) of RFP^+^ newborn neurons in the DG. GMF + Vehicle, *n* = 71 neurons from four mice, GMF + DDC, *n* = 131 neurons from four mice, HMF + Vehicle, *n* = 32 neurons from four mice, HMF + DDC, *n* = 76 neurons from four mice. Data were analyzed by UNIANOVA analysis, and the two-tailed unpaired *t* test for two-group comparisons. **m** Schematic drawing of novel location and object location test. **n**, **o** Quantification of the ratio of exploration time on novel objective location (**n**) and novel objective object (**o**) in GFM- and HMF-exposed mice with or without DDC treatment. GMF + Vehicle, *n* = 9 mice, GMF + DDC, *n* = 9 mice, HMF + Vehicle, *n* = 9 mice, HMF + DDC, *n* = 8 mice. *P* values were determined using the two-tailed unpaired *t* test. **p** Schematic drawing of fear conditioning test. **q**, **r** The percentage analysis of freezing behavior during fear conditioning tests (**q**, contextual test; **r**, cue test) in GFM- and HMF-exposed mice with or without DDC treatment, GMF + Vehicle, *n* = 9 mice, GMF + DDC, *n* = 9 mice, HMF + Vehicle, *n* = 9 mice, HMF + DDC, *n* = 8 mice. *P* values were determined using the two-tailed unpaired *t* test. The data in (**k**) were presented in the whisker plot with defined elements, median (centerline), upper and lower quartiles (bounds of box), and highest and lowest values (whiskers). Other data are presented as mean ± SEM. Tukey’s multiple comparisons test was performed to analyze the mean of each group with the mean of every other group.

As long-term exposure to HMF decreased adult hippocampal neurogenesis and reduced cellular ROS content, we then hypothesized that increasing ROS levels could rescue adult hippocampal neurogenesis. Since SOD1 is the most abundant in the nervous system^26^, we tested our hypothesis by upregulating ROS levels via diethyldithiocarbamate (DDC, an inhibitor of SOD1)^27^ administration, and assessed the proliferation and differentiation of aNSCs in HMF-exposed mice (Fig. 5a, e). We found that there were significantly higher ROS levels in aNSCs of GMF-exposed mice with DDC treatment than those without DDC treatment (Supplementary Fig. 7a, b). In contrast to GMF-exposed mice without DDC treatment, GMF-exposed mice with DDC treatment exhibited significant decreases in the numbers of BrdU^+^GFAP^+^Sox2^+^ type 1 aNSCs (Fig. 5b) and BrdU^+^GFAP^–^Sox2^+^ type 2 aNSCs (Fig. 5c) 2 h post BrdU injection. Subsequently, DDC treatment led to a significant reduction in the numbers of BrdU^+^ cells (Fig. 5f) and BrdU^+^NeuN^+^ newborn neurons (Fig. 5g), but had no significant effect on the numbers of BrdU^+^S100β^+^ astrocytes (Fig. 5h) in the DG of GMF-exposed mice 4-weeks post BrdU injection. As expected, we found that there was a significant increase in the numbers of activated caspase-3^+^DCX^+^ cells in the DG of GMF-exposed mice after DDC treatment (Fig. 5d), which is consistent with that increasing oxidative stress inhibits neurogenesis, as well as increases cell apoptosis^28^. However, DDC treatment elevated the ROS contents in aNSCs of the HMF-exposed mice back to the levels of GMF-exposed controls (Supplementary Fig. 7a, b). Furthermore, we found that HMF-exposed mice with DDC treatment exhibited increases in the numbers of BrdU^+^GFAP^+^Sox2^+^ type 1 aNSCs (Fig. 5b) and BrdU^+^GFAP^–^Sox2^+^ type 2 aNSCs (Fig. 5c) 2 h post BrdU injection, as well as the numbers of BrdU^+^ cells (Fig. 5f) and BrdU^+^NeuN^+^ newborn neurons (Fig. 5g) 4-weeks post BrdU injection. However, the numbers of BrdU^+^S100β^+^ astrocytes were not affected in HMF-exposed mice with or without DDC treatment (Fig.  5h) 4-weeks post BrdU injection. Moreover, there was a significant reduction in the numbers of Nestin-GFP^+^ cells in HMF-exposed Nestin-GFP mice after DDC treatment (Supplementary Fig. 7c, d). Thus, these data suggested that the detrimental effects of long-term HMF exposure on adult neurogenesis are reversible by a pharmacological intervention to elevate endogenous cellular ROS levels.

### Pharmacological inhibition of ROS removal rescues defective dendritic development of newborn neurons in HMF-exposed mice

We next assessed the effect of DDC administration on dendritic development of newborn neurons in both GMF-exposed and HMF-exposed mice. Although DDC treatment did not significantly reduce dendritic length (Fig. 5j, k) and dendritic complexity (Fig. 5j, l) in GMF-exposed mice, the spine development was significantly compromised (Supplementary Fig. 8a, b), which is consistent with that perturbation of oxidative stress leads to impaired neuronal integration and synaptic plasticity^28,29^. Interestingly, DDC treatment not only increased dendritic length (Fig. 5j, k) and dendritic complexity (Fig. 5j, l), but also promoted spine development of newborn neurons (Supplementary Fig. 8) in HMF-exposed mice. These data suggested that elevated ROS levels by a pharmacological intervention ameliorate defective maturation of newborn neurons in the adult hippocampus caused by HMF exposure.

### Pharmacological inhibition of ROS removal rescues defective hippocampus-dependent learning in HMF-exposed mice

Adult hippocampal neurogenesis has been known to be involved in learning and memory^14,22^. We then investigated whether long-term exposure to HMF affects hippocampus-dependent learning (Fig. 5m, p). In general, GMF- and HMF-exposed mice displayed no significant effects on locomotor activity and anxiety during the open field test (Supplementary Fig. 9). However, in contrast to GMF-exposed mice, HMF-exposed mice exhibited no preference for objects during both the novel objective location test (NOL) (Fig. 5m, n) and novel object recognition test (NOR) (Fig. 5m, o), which are dependent on intact hippocampal function and neurogenesis^30,31^. Moreover, HMF-exposed mice also showed reduced freezing behavior to contextual (Fig. 5p, q) but not cue stimuli (Fig. 5p, r) in a fear-conditioning test, in which the context test is dependent on the function of both the hippocampus and amygdala, while performance in the cue test is only dependent on the function of the amygdala^32^. These data suggested that long-term exposure to HMF impairs hippocampus-dependent learning.

Since pharmacological inhibition of ROS removal by DDC treatment rescues the defective hippocampal neurogenesis caused by HMF exposure. We then assessed whether DDC treatment could rescue the defective hippocampus-dependent learning in HMF-exposed mice. GMF- or HMF-exposed mice treated with DDC displayed no significant defects in locomotor activity or anxiety during an open field test (Supplementary Fig. 9). Compared with GMF-exposed mice without DDC treatment, however, GMF-exposed mice treated with DDC exhibited no preference for objects during either NOL test (Fig. 5m, n) or NOR test (Fig. 5m, o), as well as reduced freezing behavior to contextual text (Fig. 5p, q), but no effect on cue test (Fig. 5p, r), which is consistent with that increased oxidative stress is deleterious to cognition^28^. Interestingly, administration of DDC to HMF-exposed mice was able to increase the exploratory time towards objects in both NOL test (Fig. 5m, n) and NOR test (Fig. 5m, o). Although not affecting the performance in the cue test (Fig. 5p, r), a similar rescue effect of DDC treatment was obtained in the fear conditioning contextual test in HMF-exposed mice, which is revealed by increasing freezing behavior in the contextual test (Fig. 5p, q). Hence, these data suggested that long-term exposure to HMF impairs hippocampus-dependent learning, which can be ameliorated through a pharmacological intervention to elevate ROS levels.

### Return to GMF restores defective adult hippocampal neurogenesis in HMF-exposed mice

**Fig. 6:**
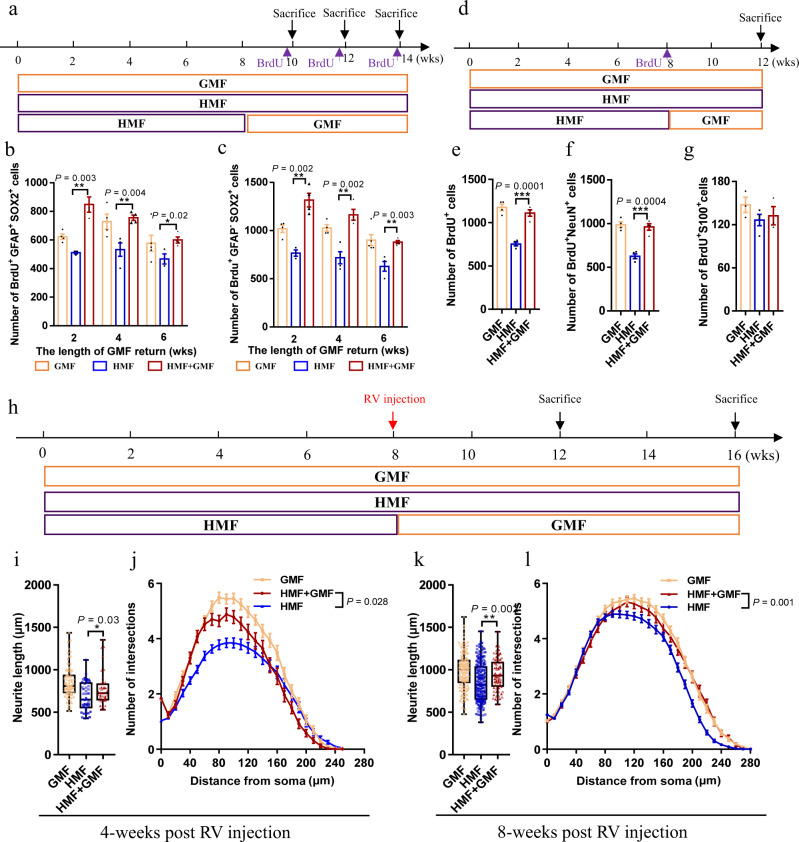
Return to GMF rescued the defective adult hippocampal neurogenesis of HMF-exposed mice. **a** Experiments timeline for cell proliferation analysis in HMF-exposed mice, followed by 2-, 4-, 6-weeks return to GMF. **b**, **c** Quantification of numbers of BrdU^+^Sox2^+^GFAP^+^ cells (**b**) and BrdU^+^Sox2^+^GFAP^−^ cells (**c**) in the DG of HMF-exposed mice with or without return to GMF. GMF, *n* = 4 mice, HMF, *n* = 4 mice, HMF + GMF, *n* = 4 mice. *P* values were determined using the two-tailed unpaired *t* test. **d** Experiments timeline for cell differentiation analysis in HMF-exposed mice, followed by 4-week return to GMF. **e**–**g** Quantification of numbers of BrdU^+^ cells (**e**), BrdU^+^NeuN^+^ cells (**f**), and BrdU^+^S100β^+^ (**g**) in the DG of HMF-exposed mice with or without return to GMF. GMF, *n* = 4 mice, HMF, *n* = 4 mice, HMF + GMF, *n* = 4 mice. *P* values were determined using the two-tailed unpaired *t* test. **h** Timeline of experiments for the in vivo labeling and analyses of newborn neurons in the DG of HMF-exposed mice, followed by return to GMF. **i**, **j** Quantification of the dendritic length (**i**) and dendritic complexity (**j**, HMF versus HMF + GMF, UNIANOVA, F(1, 120) = 4.972, *P* = 0.028) of RFP^+^ newborn neurons in the DG of HMF-exposed mice with or without return to GMF 4-weeks after retroviruses injection. GMF, *n* = 83 neurons from four mice, HMF, *n* = 50 neurons from four mice, HMF + GMF, *n* = 72 neurons from four mice. Data were analyzed by UNIANOVA analysis, and the two-tailed unpaired *t* test for two-group comparisons. **k**, **l** Quantification of the dendritic length (**k**) and dendritic complexity (**l**, HMF versus HMF + GMF, UNIANOVA, F(1, 264) = 10.944, *P* = 0.001) of RFP^+^ newborn neurons in the DG of HMF-exposed mice with or without return to GMF 8-weeks after retroviruses injection. GMF, *n* = 143 neurons from four mice, HMF, *n* = 186 neurons from four mice, HMF + GMF from four mice, *n* = 80 neurons from four mice. Data were analyzed by UNIANOVA analysis, and the two-tailed unpaired *t* test for two-group comparisons. The data in (**i**, **k**) were presented in the whisker plot with defined elements, median (centerline), upper and lower quartiles (bounds of box), and highest and lowest values (whiskers). Other data are presented as mean ± SEM. Tukey’s multiple comparisons test was performed to analyze the mean of each group with the mean of every other group.

Our data demonstrated that adult neurogenesis was significantly impaired by exposure to HMF, we, therefore, investigated whether this process is correlated with changes in the magnetic field by returning HMF-exposed mice back to GMF (Fig. 6a, d). We found that the ROS levels in aNSCs of HMF-exposed mice were reverted to the levels of GMF-exposed controls after transferring them back to GMF (Supplementary Fig. 10a, b). Interestingly, at as early as 2-weeks after returning HMF-exposed mice back to GMF, there were significant increases in the numbers of BrdU^+^GFAP^+^Sox2^+^ type 1 aNSCs (Fig. 6b) and BrdU^+^GFAP^–^Sox2^+^ type 2 aNSCs (Fig. 6c) 2 h post BrdU injection. Furthermore, 4-weeks after returning HMF-exposed mice back to GMF, the numbers of BrdU^+^ cells (Fig. 6e) and BrdU^+^NeuN^+^ neurons (Fig. 6f) were significantly increased, but the numbers of BrdU^+^S100β^+^ astrocyte were not affected (Fig. 6g), at 4-weeks post BrdU injection.

We then assessed the effect of return to GMF on dendritic development of newborn neurons in the DG (Fig. 6h). The defective dendritic length and complexity of newborn neurons in HMF-exposed mice were significantly restored at 4-weeks after returning them back to GMF (Fig. 6i, j), which were fully rescued at 8-weeks after returning them back to GMF (Fig. 6k, l).

Therefore, these data suggested that return to GMF is sufficient to rescue the deficits of aNSC proliferation and neuronal differentiation, as well as the defective maturation of newborn neurons caused by exposure to HMF.

### Return to GMF restores defective hippocampus-dependent learning in HMF-exposed mice

Because return to GMF could rescue the defective hippocampal neurogenesis in HMF-exposed mice, we then assessed whether return to GMF could rescue the defective hippocampus-dependent learning caused by exposure to HMF. There were no significant changes in locomotor activity or anxiety during the open field test in HMF-exposed mice after returning them back to GMF (Supplementary Fig. 11a–d). However, after returning back to GMF, HMF-exposed mice displayed significant increases of exploratory time towards the objects in both NOL and NOR tests (Supplementary Fig. 11e, f). A similar rescue effect of return to GMF was obtained on the fear conditioning contextual test (Supplementary Fig. 11g), but not affecting the performance in the cue test (Supplementary Fig. 11h), in HMF-exposed mice. Taken together, these data suggested that return to GMF is able to ameliorate defective hippocampal cognition caused by long-term exposure to HMF.

### Inhibition of ROS production blocks the rescue effect of return to GMF on defective adult hippocampal neurogenesis in HMF-exposed mice

**Fig. 7:**
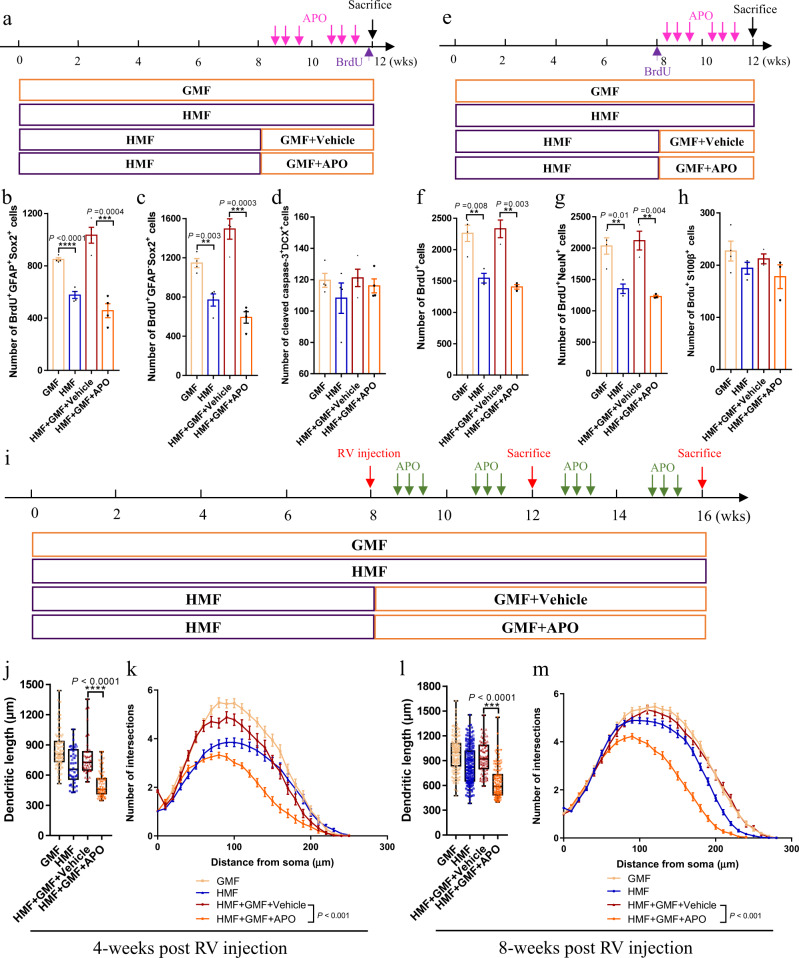
APO blocked the recovery effects of GMF return on defective adult hippocampal neurogenesis of HMF-exposed mice. **a** Experiments timeline for cell proliferation analysis in HMF-exposed mice, followed by return to GMF with or without APO treatment. **b**-**d** Quantification of numbers of BrdU^+^Sox2^+^GFAP^+^ cells (**b**) and BrdU^+^Sox2^+^GFAP^–^ cells (**c**) and active caspase-3^+^DCX^+^ cells (**d**) in the DG of HMF-exposed mice, followed by return to GMF with or without APO treatment. GMF, *n* = 4 mice, HMF, *n* = 4 mice, HMF + GMF + Vehicle, *n* = 4 mice, HMF + GMF + APO, *n* = 4 mice. *P* values were determined using two-tailed unpaired *t* test. **e** Experiments timeline for cell differentiation analysis in HMF-exposed mice, followed by return to GMF with or without APO treatment. **f**–**h** Quantification of numbers of BrdU^+^ cells (**f**), BrdU^+^NeuN^+^ cells (**g**), and BrdU^+^S100β^+^ (**h**) in the DG of HMF-exposed mice, followed by return to GMF with or without APO treatment. GMF, *n* = 4 mice, HMF, *n* = 4 mice, HMF + GMF + Vehicle, *n* = 4 mice, HMF + GMF + APO, *n* = 4 mice. *P* values were determined using two-tailed unpaired *t* test. **i** Experiments timeline for the in vivo labeling and analyses of newborn neurons in HMF-exposed mice, followed by return to GMF with or without APO treatment. **j**, **k** Quantification of the dendritic length (**j**) and dendritic complexity (**k**, HMF + GMF + Vehicle versus HMF + GMF + APO, UNIANOVA, F(1, 150) = 29.23, *P* < 0.001,) of RFP^+^ newborn neurons in the DG of HMF-exposed mice, followed by return to GMF with or without APO treatment, 4-weeks after retroviruses injection. GMF, *n* = 82 neurons from four mice, HMF, *n* = 46 neurons from four mice, HMF + GMF + Vehicle, *n* = 72 neurons from four mice, HMF + GMF + APO, *n* = 80 neurons from four mice. Data were analyzed by UNIANOVA analysis, and the two-tailed unpaired *t* test for two-group comparisons. **l**, **m** Quantification of the dendritic length (**l**) and dendritic complexity (**m**, HMF + GMF + Vehicle versus HMF + GMF + APO, UNIANOVA, F(1, 209) = 138.45, *P* < 0.001) of RFP^+^ newborn neurons in the DG of HMF-exposed mice, followed by return to GMF with or without APO treatment, 8-weeks after retroviruses injection. GMF, *n* = 144 neurons from four mice, HMF, *n* = 187 neurons from four mice, HMF + GMF + Vehicle, *n* = 80 neurons from four mice, HMF + GMF + APO, *n* = 131 neurons from four mice. Data were analyzed by UNIANOVA analysis, and the two-tailed unpaired *t* test for two-group comparisons. The data in (**j**) were presented in whisker plot with defined elements, median (centerline), upper and lower quartiles (bounds of box), and highest and lowest values (whiskers). Other data are presented as mean ± SEM.

**Fig. 8:**
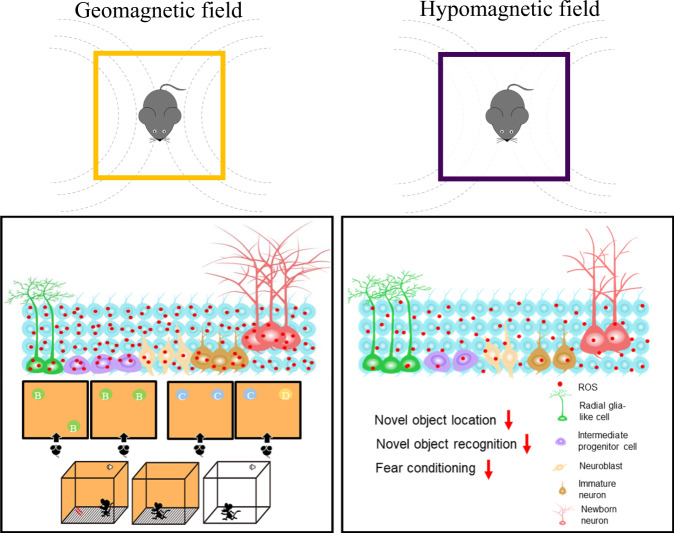
Working model illustrating the effects of GMF and HMF on adult hippocampal neurogenesis. GMF regulates hippocampal neurogenesis and hippocampus-dependent learning through maintaining proper endogenous ROS levels (red dots). However, HMF attenuates hippocampal neurogenesis and hippocampus-dependent learning by reducing endogenous ROS levels.

NADPH oxidases (NOX)-mediated ROS production is one of the major cellular sources for ROS^33^. Apocynin (APO, 4′-hydroxy-3′-methoxy-acetophenone) inhibits the NOX enzymes by acting on the translocation of the cytoplasmic subunits of the enzymes^34^. To investigate whether the rescue effects of return to GMF on defective adult hippocampal neurogenesis is correlated with increases of endogenous ROS levels, we then injected APO to inhibit ROS production when returning HMF-exposed mice back to GMF (Fig. 7a, e). We found that the promoting effect of return to GMF on ROS levels in aNSCs of HMF-exposed mice was fully blocked after APO treatment (Supplementary Fig. 10a, b). Moreover, APO treatment prevented the rescue effects of return to GMF on the numbers of BrdU^+^GFAP^+^Sox2^+^ type 1 aNSCs (Fig. 7b) and BrdU^+^GFAP^–^Sox2^+^ type 2 aNSCs (Fig. 7c) in HMF-exposed mice 2 h post BrdU injection, as well as the numbers of BrdU^+^ cells (Fig. 7f) and BrdU^+^NeuN^+^ neurons (Fig. 7g) in HMF-exposed mice 4-weeks post BrdU injection. In addition, we found that return to GMF significantly decreased the number of GFP^+^ aNSCs in HMF-exposed Nestin-GFP mice, while APO treatment blocked such decreasing effect (supplementary Fig. 10c). Furthermore, APO treatment also impeded the restoring effect of return to GMF on defective dendritic development of newborn neurons in HMF-exposed mice at 4 weeks (Fig. 7i–k) and 8 weeks (Fig. 7i, l, m) post retroviruses injection respectively. However, APO treatment did not affect cell apoptosis (Fig. 7d) and astrocytic differentiation (Fig. 7h) in the DG of HMF-exposed mice when returning to GMF. Therefore, our data suggested that adult hippocampal neurogenesis could respond to the changes of the magnetic field by modulating cellular ROS levels.

### Inhibition of ROS production blocks the restoring effect of return to GMF on defective hippocampus-dependent learning in HMF-exposed mice

We then assessed whether the rescue effect of return to GMF on defective hippocampus-dependent learning in HMF-exposed mice could be prevented by APO treatment. In general, APO treatment did not affect the locomotor activity or anxiety in HMF-exposed when returning to GMF (Supplementary Fig. 12a–d), However, APO treatment was able to prevent the restoring effect of return to GMF on the exploratory time towards objects in both NOL and NOR tests (Supplementary Fig. 12e, f) in HMF-exposed mice. In addition, APO treatment blocked the rescuing effect of return to GMF on the freezing behavior to contextual test (Supplementary Fig. 12g), but not affected the performance on cue test (Supplementary Fig. 12h), in HMF-exposed mice. Thus, these data suggested that the effects of magnetic fields on hippocampus-dependent learning are correlated with endogenous ROS levels.

## Discussion

Our data here demonstrated that long-term HMF exposure impairs adult hippocampal neurogenesis and cognition through decreasing endogenous cellular ROS concentrations (Fig. 8). High ROS levels have been known to be associated with oxidative stress, aging, brain dysfunction^29,35,36^. However, low ROS levels, on the other hand, impair learning and memory, cell proliferation and migration^24,37–39^. In our study, we found that exposure to HMF led to reduced ROS levels and inhibited neurogenesis, subsequently impaired hippocampus-dependent learning. Furthermore, we found that inhibition of CuZn-SOD activities by DDC treatment significantly elevated ROS in HMF-exposed mice back to the level of GMF-exposed mice, and rescued defective hippocampal neurogenesis and hippocampus-dependent learning in HMF-exposed mice. But the administration of DDC to GMF-exposed mice led to significant increases in ROS levels, and impaired adult neurogenesis as well, which are consistent with previous studies that CuZn-SOD deficiency leads to a severe reduction in neuronal production in adult hippocampus^40,41^. Moreover, we found that returning HMF-exposed mice back to GMF not only elevated ROS levels, but also rescued defective neurogenesis and hippocampus-dependent learning caused by exposure to HMF. Whereas, these rescue effects of return to GMF in HMF-exposed mice were fully blocked after inhibition of NOX by APO treatment. APO can not only serve as a NOX inhibitor that inhibits endogenous superoxide/hydrogen peroxide production but also acts as a general antioxidant by reacting with ROS^42^. On the other hand, genetic deletion of NADPH oxidase has been shown to impair neurogenesis and neuronal development^43^. In summary, maintenance of appropriate levels of ROS by GMF is required for proper adult hippocampal neurogenesis and function.

Although the effect of magnetic field on ROS levels is still controversial^44^, HMF has been found to decrease cellular ROS levels^45,46^. In our study, we quantified ROS levels by hydroethidine dye, which only detects superoxide but not all ROS. Therefore, it will be interesting to use a genetically encoded ROS-biosensor to the ROS content, such as superoxide and hydrogen peroxide, in the further study. Nonetheless, we observed that long-term exposure to HMF decreased endogenous ROS concentrations in aNSCs, which is consistent with previous findings. However, the underlying mechanism of magnetic field modulating ROS expression remains unclear. Several chemical magnetoreception and quantum magnetoreception hypotheses, e.g., radical pair^47^, universal physical mechanism^6^, magnetic nanoparticles^48^, and water proton^49^, have been proposed for explanation of biological effects of HMF. Among them, the radical pair-based mechanism proposes that the singlet/triple interconversion rates of unpaired radicals formed in the course of cryptochrome redox chemistry can be altered by static magnetic fields^47^. However, the biological effects caused by radical pair-based mechanism may be tentatively overlooked here, because it usually requires a magnetic field larger than 30 μT^6^, not the case of the present study (HMF, 0.29 ± 0.01 μT). Moreover, cryptochromes have been characterized for a role as core components of the circadian clock in mammalian cells, although they have been proposed to function independently in their role of the circadian clock as magnetoreceptor^50^. However, it cannot completely exclude the possibility of circadian rhythms-induced oxidative stress by cryptochromes^51^. In addition, mitochondrial activity has been found to be declined in HMF^52^, but mitochondrial is not the unique source for ROS production^53^. Nevertheless, most studies on ROS have focused on their cellular toxicity; however, recent studies have suggested that ROS may act as a physiological signaling molecule or regulator to govern neuronal development and function^54^. Therefore, it would be interesting to decipher the mechanisms underlying ROS response to the magnetic field, and ROS-regulated brain development and function.

In deep space, the combined effects of different space flight factors on humans are the most important and urgent problems to be solved in astrobiology research. Among them, HMF and space ionizing radiation are two main space flight factors that significantly cause acute and chronic CNS risks defeats, and they might affect the operating activity of astronauts during deep space missions^5,12^. Radiation not only elevates ROS levels, leading to the formation of many toxic products in free radical reactions to trigger neuroinflammation^55^ but also impedes hippocampal neurogenesis and impairs hippocampus-dependent cognitive abilities^56,57^. These studies are at dose rate much higher than in deep space (~0.5 mGy/d at solar minimum)^58^. Moreover, the significant changes in neurogenesis and neuroinflammation have not been found at a dose of 0.5 Gy of ^56^Fe radiation^59^. In addition, antioxidant treatment did not prevent radiation-induced impairments in hippocampal-dependent learning, suggesting that the detrimental effect of irradiation on cognition might undergo a mechanism unlikely involving ROS or oxidative damage^57^. However, the detection of DNA damage induced by radiation was correlated with space flight duration^60^, suggesting the deleterious effects of radiation could be accumulated during space flight. In our study, it was showed that long-term exposure to HMF impedes adult hippocampal neurogenesis and cognition by decreasing endogenous ROS concentrations. Although space radiation and HMF are two major environmental stressors encountered in space travel, the interplay of space radiation and HMF in ROS and ROS-regulated neurogenesis and cognitive processes is still unknown. Therefore, it would be very interesting to carefully design experiments to solve this puzzle.

## Methods

### Magnetic field setups

The experiments for animals were carried out in a basement room with a constant temperature and relative humidity (22 ± 1 °C; 65 ± 1%). The homogeneous HMF was generated by a custom-made tri-axial square Helmholtz coil system as described previously^21^. Briefly, two identical tri-axial Helmholtz coil systems are composed of three pairs of orthogonally aligned 1.5-m square coils, in which the number of wire loops in the X, Y, and Z axes is 190, 114, and 228, respectively, were used to generate the system. The HMF was generated by simultaneously modulating the DC power generator of the X, Y, and Z axes to generate an HMF in each specific axis. The coil system provided a uniform net magnetic field space in the coil center (more than 50 × 50 × 50 cm). In particular, a 4-layer wooden shelf with open extremities (length, 55 cm; width, 55 cm; and height, 110 cm; height interval, 30 cm; the height of the lowest layer, 20 cm) was positioned in the center of tri-axial square Helmholtz coil system which allowed to position the transparent plastic cages containing freely moving mice in the middle two layers with a uniform net magnetic field space. The Helmholtz coil system is covered with invisible black fabric around and lights are installed on the top of the system with a 12-h reverse light/dark cycle. All cages were of the same size (27 × 16 × 13 cm), equipped with plastic lids and water bottles. For the GMF control, the same apparatus was used, but the DC power generator was off. To insulate mice from noise generated from the DC power of the Helmholtz coil system when it is on, the DC power is placed in a soundproof room next to the one where the Helmholtz coil system is set up (Supplementary Fig. 1a).

The magnetic strength inside the cages of HMF and GMF environment was monitored by the APS 520 3-axis fluxgate magnetometer (Applied Physics Systems, Mountain View, California) every three days for 150 days (Supplementary Fig. 1b). The ambient electromagnetic field inside the Helmholtz coils systems was measured by a Digital Fluxgate Magnetometer (Magson GmbH, Berlin, Germany) (Supplementary Fig. 1c and Supplementary Table 2). The noise levels inside the Helmholtz coil system were measured by an NTI Audio XL2 Audio and Acoustic Analyzer (NTI-XL2, NTi Audio, Swiss) (Supplementary Fig. 1d). The light intensity inside the Helmholtz coil system was measured by a light meter (941, Fluke, USA) (Supplementary Fig. 1e).

For aNSCs analysis in vitro, aNSCs were cultured in a permalloy magnetic shielding chamber in a CO_2_ incubator in the Institute of Biophysics, Chinese Academy of Sciences, as the described previously^46^. Briefly, the CO_2_ incubator was divided into two identical apartments, the upper apartment for GMF, the lower apartment for HMF. The 95% relative humidity, 37 °C and 5% CO_2_ concentration were set in both incubators. The magnetic fields in the HMF magnetic shielding box and GMF’s cell incubator were also measured by the Fluxgate Magnetometer and FFT algorithm was used to calculate the square root of PSD of the AC magnetic field, as seen in Supplementary Table 2. The residual static magnetic field inside the magnetic shielding chamber was about 0.17 μT. For GMF control, the cell incubator chamber with a magnetic field of 39.4 ± 3.6 μT.

### Mice

Adult male mice (C57BL/6 J) were housed in the animal facility with a constant temperature and relative humidity (22 ± 1 °C; 65 ± 1%) at the Institute of Genetics and Developmental Biology (IGDB), Chinese Academy of Sciences, on a 12-h reverse light/dark cycle with lights on at 08:00 am and off at 08:00 pm. All procedures and husbandry were performed according to protocols approved by the Institutional Animal Care and Use Committee at IGDB. Adult male mice (8–10 weeks old) on a C57BL/6 J background were used. Nestin-GFP mice^61^ were backcrossed to the C57BL/6 J background for at least six generations. The mice were randomly allocated to experimental groups: (i) GMF exposure and (ii) HMF exposure. The mice were group-housed and four mice per cages. Cages were changed once a week in both apparatuses. The detailed information about treatments on mice is in Supplementary Table 1. Animals were randomly allocated to experimental groups, and the experimenter was blinded to treatment groups of the animals until the experiment was complete. No data or animals were excluded from the analysis. All groups with different treatments were run simultaneously.

### BrdU administration

For the in vivo aNSCs proliferation assay, the mice received bromodeoxyuridine (BrdU) injections (Sigma-Aldrich, B5002, 200 mg/kg) and were sacrificed 2 h post-BrdU injection. For the in vivo aNSCs differentiation assay, the mice received BrdU injections (100 mg/kg/day) for 4 days and were sacrificed at 1, 2, or 4 weeks post BrdU injection.

### Diethyldithiocarbamate (DDC) administration

Systemic administration of sodium diethyldithiocarbamate trihydrate (DDC, an inhibitor of SOD1 activity, Sigma-Aldrich, 228680, 400 mg/kg in phosphate-buffered saline, PBS)^27^ was performed by daily intraperitoneal injections for 4 weeks. PBS administration was used as a vehicle control.

### Apocynin (APO) administration

Systemic administration of Apocynin (Apo, the NADPH oxidase inhibitor, Sigma-Aldrich, 178385, 20 mg/kg in DMSO)^24^ was performed by daily intraperitoneal injections for 4 weeks. DMSO administration was used as vehicle control.

### Dihydroethidium (HEt) administration

Administration of the ROS-sensitive dye dihydroethidium (HEt, 25 mg/kg; Sigma-Aldrich, 37291)^24^ was performed by intraperitoneal injection 4 h prior to perfusion fixation. The brain sections were analyzed for fluorescent intensity using ImageJ software (NIH, Bethesda, Maryland, USA).

### Tissue preparation and immunohistochemistry

Mice were euthanized by intraperitoneal injection with Avertin and perfused transcardially with saline followed by 4% paraformaldehyde (PFA). Brains were dissected and post fixed in 4% PFA overnight and then equilibrated in 30% sucrose buffer. Brains were sectioned in the coronal plane on a freezing microtome at a thickness of 40 µm. Serial sections of the hippocampus were stored in 96-well plates filled with cryoprotectant solution (glycerol, ethylene glycol, and 0.1 M phosphate buffer, pH 7.4, 1:1:2 by volume) in a −20 °C freezer.

The tissue sections (40-μm thick) prepared from injected mice were pre-blocked with blocking buffer TBS^++^ (TBS containing 3% donkey serum and 0.3% Triton X-100) for 1 h at 37 °C, followed by incubation with primary antibodies diluted in blocking buffer for 24 h at 4 °C. After washing three times, sections were incubated with the secondary antibody for 1 h at 37 °C. To detect BrdU incorporation, tissue sections were pretreated with 2 M HCl for 20 min at 37 °C, incubated with borate buffer (pH 8.5) for 30 min, and subjected to immunocytochemistry analyses. All sections were counterstained with 4′,6-diamidino-2-phenylindole dihydrochloride (DAPI, Sigma-Aldrich, #B2261). The primary antibodies used were as follows: rat anti-BrdU (1:1000, Abcam, ab6326), goat anti-GFAP (1:1000, Abcam, ab53554), mouse anti-Sox2 (1:1000, Abcam, ab79351), rabbit anti-Ki67 (1:500, Invitrogen, MA5-14520), mouse anti-NeuN (1:500, Millipore, MAB377), rabbit anti-S100β (1:1000, Dako, Z0334), goat anti-DCX (1:100, Santa Cruz Biotechnology, SC-8066), rabbit anti-ki67 (1:500, Abcam, ab15580), and rabbit anti-cleaved caspase-3 (1:500, Cell Signaling Technology, #96644). The following fluorescent secondary antibodies were used: donkey anti-rat Alexa Fluor 488 (1:500, Invitrogen, A21208), donkey anti-goat Alexa Fluor 568 (1:500, Invitrogen, A11057), donkey anti-mouse Alexa Fluor 647 (1:500, Invitrogen, A31571), donkey anti-rabbit Alexa Fluor 488 (1:500, Invitrogen, A21206), donkey anti-rabbit Alexa Fluor 647 (1:500, Invitrogen, A31573), donkey anti-mouse Alexa Fluor 568 (1:500, Invitrogen, A10037), donkey anti-goat 647 (1:500, Invitrogen, A21447), and donkey anti-rabbit Alexa Fluor 568 (1:500, Invitrogen, A10042). After staining, sections were mounted, coverslipped, and then maintained at 4 °C in the dark until imaging. Images were acquired on an Olympus FV1000 multiphoton confocal system with a multitrack configuration.

### Quantification and fate mapping of BrdU^+^ cells in the DG

For quantification of BrdU^+^ cells, Ki67^+^ cells, the phenotype of BrdU^+^ cells (double labeling with either GFAP, Sox2, Ki67, DCX, S100β, or NeuN) and Nestin-GFP cells, 1 in 12 serial sections starting at the beginning of the hippocampus (relative to bregma, −1.5 mm) to the end of the hippocampus (relative to bregma, −3.5 mm) were used. All indicated cells in the brain sections (4–6 sections per mouse) were counted inside the section center between 5-μm guard zones of the section surfaces under a Nikon-ECLIPSE 80i microscope with NIS-Elements, BR. 3.00 software^62^. The data were presented as a number of cells in a cubic millimeter of the dentate gyrus.

For measurement of relative DG volume, 1 in 12 serial sections starting at the beginning of the hippocampus (relative to bregma, −1.5 mm) to the end of the hippocampus (relative to bregma, −3.5 mm) were stained with DAPI to visualize the nuclei. The same amount of sections (4 sections per mouse) with relative same brain anatomy was used to measure granule cell layer volume under a Nikon-ECLIPSE 80i microscope with NIS-Elements, BR. 3.00 software, and the data were presented as relative DG volume among the selected sections.

### Recombinant retrovirus production

Retrovirus production was performed as described previously^63^. Briefly, viral transfer vector DNA and packaging plasmid DNA were transfected into cultured 293T cells using calcium phosphate methods. The medium was collected and pooled at 40, 64, and 88 h and then filtered through a 0.2 μm filter. Viruses were concentrated by ultracentrifugation at 19,000 g for 2 h at 20 °C using an SW27 rotor (Beckman). The virus was washed once with phosphate-buffered saline (PBS) and then resuspended in 150 μL PBS. We routinely obtained ~1 × 10^9^ infectious viral particles/mL.

### In vivo retrovirus grafting

In vivo retrovirus grafting was performed as described^63^. C57BL/6 J male mice were anesthetized with isoflurane, and the retrovirus expressing red fluorescent protein (RFP) (1 μL with a titer greater than 5 × 10^8^/mL) was stereotactically injected into the dentate gyrus using the following coordinates relative to bregma: caudal: −2.0 mm; lateral: +/−1.7 mm; ventral: −1.9 mm. The RFP^+^ neurons around this relative bregma area, but not in the injection site, were used.

### Dendritic morphology analyses

For the dendritic branching analysis on 100-μm thick floating brain sections, the retrovirally infected neurons were imaged on an LSM 550 confocal microscope with a 20× oil objective 3D reconstruction of entire dendritic processes of each neuron in the dentate gyrus was made from Z-series stacks of confocal images at 3-μm intervals. The 2D projection images were imported and traced by NIH ImageJ software with a sholl analysis plugin (https://imagej.net/Sholl). Data were extracted for Sholl analysis and total dendritic length from each retrovirally infected neuron. Roughly 20–50 neurons with largely intact dendritic trees per mouse were traced, and at least four different animals per group were analyzed. The exact value of *n* is described in the figure legends.

### Fluorescence-activated cell sorting

Nestin-GFP mice were sacrificed by cervical dislocation, and their brains were immediately dissected out and placed in ice-cold HBSS. The hippocampus was dissected and digested with papain (LK003178 Code PAP2, Worthington Biochemical) based on published methods. After digestion and rinsing, the tissues were gently filtered with a 70-mm cell strainer. DAPI was added to the cell suspension prior to sorting to exclude dead cells. Cell suspensions were sorted on a FACSAriaII (BD) using an 80-μm nozzle at 20 psi. GFP^+^ cells were directly sorted into ice-cold cell lysis buffer (Triton X-100 containing RNAse inhibitors, Berry Genomics) and stored at −80 °C until library preparation.

### Library preparation, sequencing, and gene ontology analysis

RNA-seq libraries were prepared using Smart-seq2 technology based on previously described methods^64^. Briefly, lysed cells were thawed and subjected to the SMART-Seq v4 Ultra Low Input RNA Kit (Takara) as per the manufacturer’s recommendations, using 18 cycles of amplification. Libraries were made using the Nextera XT kit (Illumina) and individually cleaned with 0.8× AMPure XP SPRI beads (Beckman Coulter) after the PCR step. Libraries were quantified using the Quant-IT DNA High-Sensitivity Assay Kit (Invitrogen), examined using a high sensitivity DNA chip (Agilent), and then pooled at equal concentrations for sequencing. Finally, deep sequencing of samples (20 million reads per cell) was performed using a Nova6000 sequencer (Berry Genomics).

### Processing and analysis of the RNA-seq data

The quality of the raw reads was checked by FASTQC, and reads were trimmed using Btrim64. Trimmed reads were mapped to the mouse genome (ENSEMBL Release 78) using tophat2 and to mouse genes using RSEM. The expression level of each gene was quantified in units of transcript per million (TPM), and fragments per kilobase of transcript per million fragments were mapped (FPKM) using RSEM (V1.2.15) with the default parameters. To compare the expression levels of different genes across samples, an additional TMM (trimmed mean of *M* values) normalization on FPKM using Trinity based on edgeR was performed. Differential expression analysis was performed with DESeq2 with a two-fold change and FDR < 0.1 (Berry Genomics). Gene Ontology analysis was performed separately on the downregulated and upregulated genes using the DAVID Bioinformatics Functional Annotation Tool^64^.

### Isolation and culture of adult NSCs

The adult neural stem/progenitor cells (aNSCs) used in this study were isolated from the dentate gyrus of 8-week-old male mice based on published methods^65^. Briefly, 8-week-old male mice were sacrificed by cervical dislocation, and their brains were immediately dissected out and placed in ice-cold Hank’s balanced salt solution (HBSS, Gibco, 14025076). The hippocampus was dissected and digested with papain (LK003178 Code PAP2, Worthington Biochemical). DMEM/F-12 medium containing 10% fetal bovine serum (FBS) was used to stop the digest. After rinsing with initial proliferation medium (IPM) and spinning down, the cell pellets were resuspended with the same IPM (neurobasal medium containing 2% B27 (Gibco, 17504-044), 2 mM L-glutamine (Gibco, 25030081), 20 ng/ml basic fibroblast growth factor (FGF-2, PeproTech, K1606), 20 ng/ml epidermal growth factor (EGF, Pepro-Tech, A2306), and 1% antibiotic–antimycotic (Gibco, 15240062)), plated into one well of a 24-well dish, and cultured in a 5% CO_2_ incubator at 37 °C. Half of the medium was replaced every 2 days.

For HMF treatment, aNSCs were cultured in a permalloy magnetic shielding chamber in a CO_2_ incubator as described previously^46^. The residual static magnetic field inside the magnetic shielding chamber was less than 0.2 μT. For GMF control treatment, aNSCs were cultured outside the magnetic shielding chamber with a magnetic field of 39.4 ± 3.6 μT. The fluxgate magnetometer and the FFT algorithm of power spectral density in the HMF magnetic shielding box and GMF’s cell incubator were presented in Supplementary Table 2.

### Western blot

Cells were lysed with a homogenizer in ice-cold Triton X-100 lysis buffer (20 mM Tris-HCl, pH 7.5, 100 mM NaCl, 1% Triton X-100, 1 mM phenylmethanesulfonylfluoride, PMSF) containing Complete Protease Inhibitor Cocktail (Roche, 11697498001). The cell lysates were centrifuged at 12,000 g for 10 min. The supernatants were mixed with sodium dodecyl sulfate-polyacrylamide gels (SDS-PAGE). Then, 20 µg of protein from each sample was separated on a 15% Tris-HCl denaturing gel by SDS-PAGE, transferred to a nitrocellulose membrane for 2 h at 120 V, blocked with 5% bovine serum albumin (BSA), and incubated with primary antibodies for 24 h at 4 °C. The membranes were incubated with HRP-conjugated goat anti-rabbit or mouse secondary antibodies for 2 h at room temperature. Rabbit anti-SOD1 (1:500, Abcam, ab13498) was the primary antibody, while rabbit anti-β-Actin (1:1000, Abcam, ab8227) was used as the internal control.

### Enzyme-linked immunosorbent assay (ELISA)

The cellular SOD1, SOD2, and SOD3 contents in cultured aNSCs of the DG were measured with SOD1 and SOD2 ELISA kits (Cusabio, Wuhan, China), respectively. Cells were seeded in 100 mm Petri dishes and cultured in a CO_2_ incubator as described above. Samples were harvested after 48 h of growth in the HMF and GMF conditions. Cells were resuspended in 300 μL of cold DPBS and mechanical disrupted. The protein concentration of each sample was determined by a bicinchoninic acid (BCA) protein assay kit according to the manufacturer’s instructions (Pierce/Thermo Fisher Scientific, USA). All ELISA procedures were in accordance with the manufacturer’s instructions.

### SOD activity assay

aNSCs were harvested after 48-h culture in the HMF and GMF conditions. The activity of total SOD (CuZn/Mn) and Mn-SOD in cultured aNSCs were measured with a total SOD assay kit and Mn-SOD assay kit (Beyotime, China), respectively, according to the manufacturer’s instructions.

### RNA isolation and quantitative real-time PCR

RNA isolation from cells or brain tissue was performed using Trizol (Invitrogen, 15596026) according to the manufacturer’s instructions. First-strand cDNA was generated by reverse transcription (A5001, Promega). RT-qPCR was performed with the QuantStudio Real-Time PCR system (Applied Biosystems). The primers used for qPCR were listed in Supplementary Table 3.

### Behavioral tests

#### Open-field test

The open-field test was performed as previously described^62^. Mice were placed in an empty white chamber box with clear sidewalls (10 × 10 × 16 in.; RWD life Science) and allowed to freely explore the box for 10 min. They were then returned to their home cages after the test. Their locomotor activity was recorded by photo beams preinstalled in the box and then analyzed with Panlab SMART 3.0 Software.

#### Novel object recognition (NOR) or novel object location recognition (NOL) test

The novel object and novel object location recognition tests were performed as previously described^31^. Briefly, mice were habituated in an empty white chamber box for 15 min. After 24 h, each mouse was returned to the same chamber with two identical objects placed in the corners and allowed to freely explore until 30 s were spent exploring both objects. After 24 h, object recognition was tested by substituting a novel object or location, which was counterbalanced in the chamber; the exploratory behavior of the mice was recorded for 10 min using TV/VCR. The time spent exploring each object was recorded using Corel VideoStudio Pro X5. Novel object or location preference is expressed as the percentage of time spent exploring the novel object or location compared with the cumulative time spent exploring both objects.

For the NOR and NOL test, two identical big clips with 3.5 inch height were used for NOL, two identical metallic cones with 3.5 in. height, and dolls with 5 in. height were used for NOR. The same groups of mice undergo every test. NOL test was performed firstly, the novel object recognition experiment was then carried out 4 days later.

#### Fear conditioning test

The fear conditioning test was performed as described^32^. Mice were habituated to a shock chamber for 2 min and then received a mild footshock (0.5 mA) (unconditioned stimulus or “US”) during the last 1.5 s of a 30-s white noise tone (87 dB) (conditional stimulus or “CS”). Two minutes later, mice received the same tone-footshock pairing (CS–US). The cycle was performed three times. After 24 h, the context test was performed by placing the mice back into the same chamber and monitoring them for 5 min. The cue test was performed 2 h later, in which colored plexiglass inserts were used to change the chamber surrounding. Mice were placed in and monitored for 6 min with two CS (administered in the same way as in the training session) given in the meantime. All events were programmed in the fear conditioning test, and the data were recorded using the Startle and Fear conditioning system (Panlab) and Packwin software (V2.0.05).

### Statistical analyses

All experiments and data analyses were conducted in a blinded manner, including the immunohistochemistry, dendritic morphology analyses, ELISA, and behavioral analyses. Data were analyzed with Graphpad Version 6.01, Image J-win64, Olympus FV10-ASW 2.0 Viewer, Adobe Illustrator CS6 (version 16), Adobe Photoshop CC 2015, Microsoft office 2011, IBM SPSS_22.0 software.

For data analysis, data were performed using ANOVA or Student’s *t* test, unless otherwise specified, with the aid of SPSS version 22. Statistical comparisons between the two groups were made using two-tailed Student’s *t* test. For cell fate analysis of HMF- and GMF-exposed mice in vivo at a different time point, two-way ANOVA were used. Sholl analysis was carried out using a univariate analysis of variance (UNIANOVA) with SPSS statistical software. All data were shown as the means with standard errors of the mean (means ± SEM). Probabilities of *p* < 0.05 were considered significant with asterisks in figures denoting *P* values as follows: *P < 0.05, **P < 0.01, ***P < 0.001, ****P < 0.0001. No statistical methods were used to predetermine sample sizes, but our sample sizes are similar to those reported in previous publications^62,63^. Supplementary Table 1 provided the data of the sample sizes for all experiments.

### Reproducibility

Experiments were repeated independently with similar results at least three times. Micrographic images presented in figures are representative ones from experiments repeated independently. Figure 1c (three times), Fig. 1e, g, i (four times), Fig. 2c, e, h, k (three times), Fig. 3c (three times), Fig. 4f, h (four times), Fig. 5j (three times), Supplementary Fig. 2b (three times), Supplementary Fig. 5d (three times), Supplementary Fig. 6a (three times), Supplementary Fig. 7a (three times), Supplementary Fig. 7c (four times), Supplementary Fig. 8a (three times), Supplementary Fig. 10a (four times).

### Reporting summary

Further information on research design is available in the Nature Research Reporting Summary linked to this article.

## Supplementary information

Supplementary Information

Reporting Summary

## Data Availability

Source data are provided with this paper. All data are contained in the main text and the supplementary materials, and are provided as a Source Data file. Source data are provided with this paper.

## Source data

Source Data

## Code availability

All raw sequenced data are stored in the Sequence Read Archive (SRA) under BioProject accession PRJNA643878. (https://www.ncbi.nlm.nih.gov/Traces/study/?acc=PRJNA643878).
