# Supplementary material for: Long-term exposure to a hypomagnetic field attenuates adult hippocampal neurogenesis and cognition
Source: Nat Commun. 2021 Feb 19;12:1174. doi: 10.1038/s41467-021-21468-x (PMC7896063; doi:10.1038/s41467-021-21468-x)
Supplement: Supplementary file 1 — Supplementary Information [file 41467_2021_21468_MOESM1_ESM.pdf]

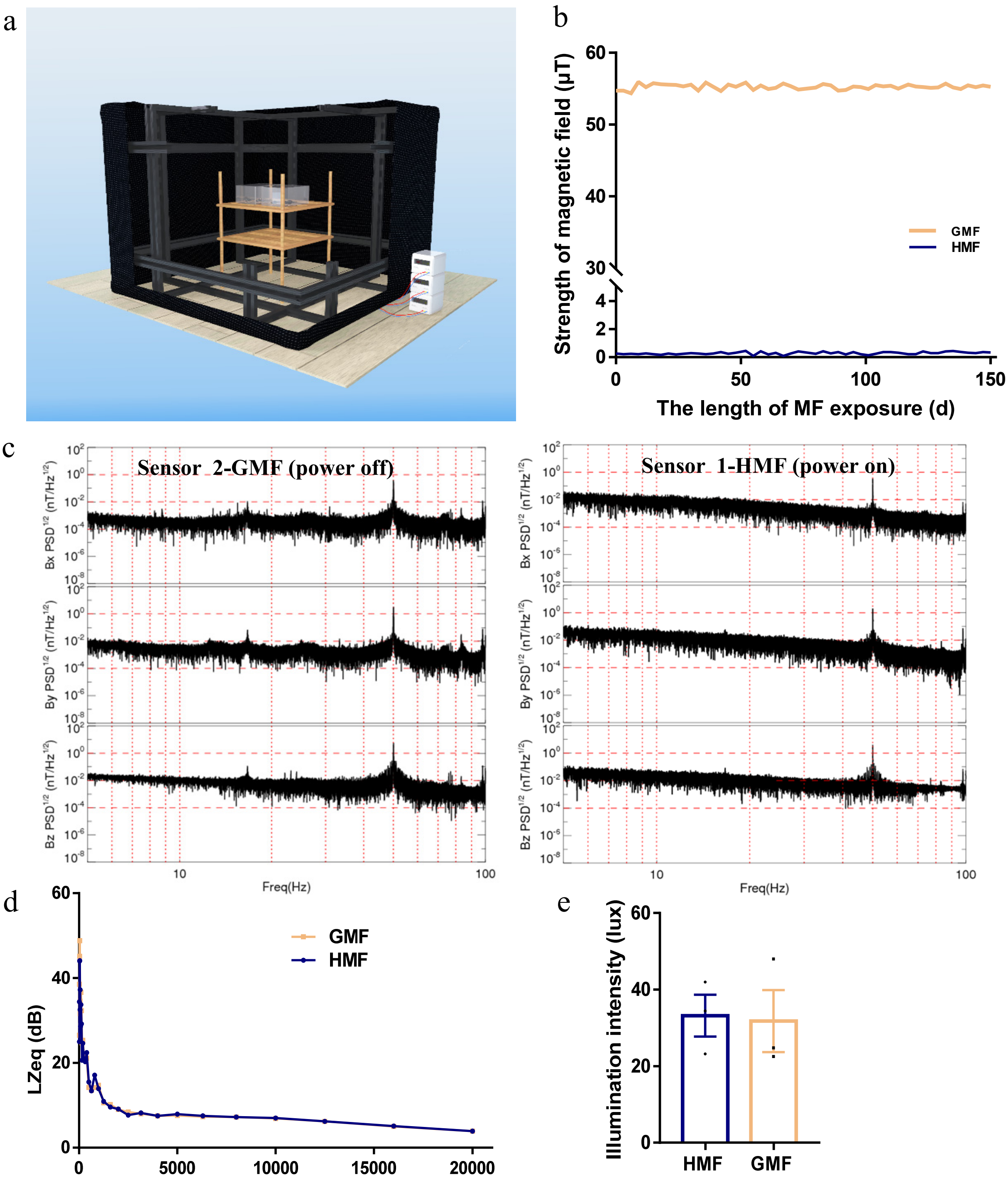

Supplementary Fig. 1. Experimental apparatus and environmental parameters in GMF and HMF.

(a) Schematic diagram of the experimental apparatus for HMF. (b) Magnetic field intensity of GMF and HMF in a150-days experimental duration. (c) The square root of power spectral density (PSD) of the ambient magnetic field at frequencies ranging from 5Hz to 100 Hz inside the cages of GMF environment with power off and HMF environment with power on. (d) The background noise level inside the cages of GMF and HMF environment. (e) The light illumination intensity inside the cages between GMF and HMF environments (GMF versus HMF, P = 0.892, two-tailed unpaired t-test), GMF, n = 3, HMF, n = 3. Data are presented as mean ± SEM, and error bars were presented in SEM.

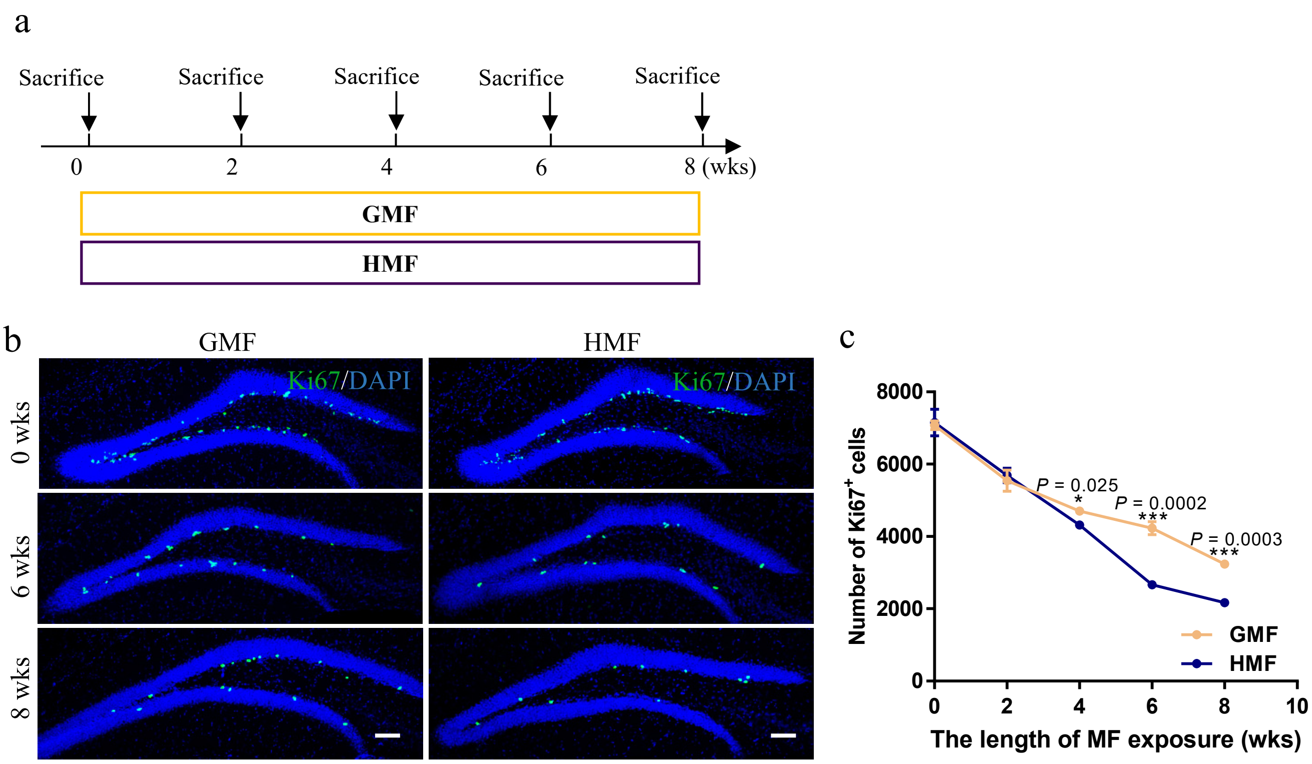

Supplementary Fig. 2. Long-term exposure to HMF decreased the cell proliferation in the DG of mice.

(a) Experiments timeline for cell proliferation analysis during HMF or GMF exposure. (b) Representative images of $Ki67^+$ cells in the DG of mice at 0-, 6-, 8-weeks GMF- or HMF-exposure. Scale bar = 200 μm. (c) Quantification of numbers of $Ki67^+$ cells in the DG of GMF- and HMF-exposed mice (GMF, n = 4 mice, HMF, n = 4 mice, GMF versus HMF, two-way ANOVA, F(1, 30) = 22.1, $P < 0.0001$). All data are presented as mean ± SEM, and error bars were presented in SEM. Data were analyzed by two-way ANOVA, and the two-tailed unpaired *t*-test for two group comparisons at each time-point.

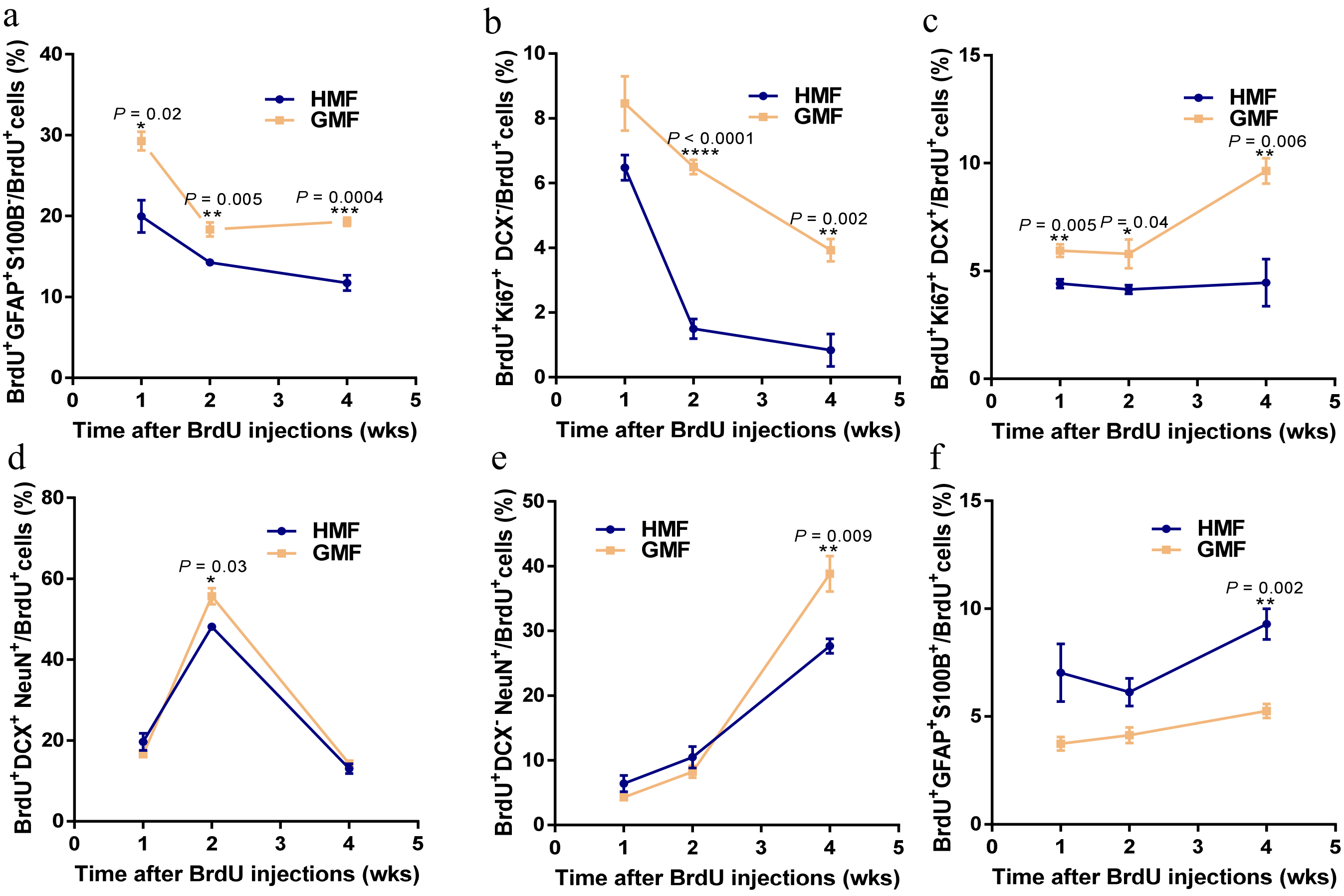

Supplementary Fig. 3. Long-term exposure to HMF altered cell lineage progression during adult hippocampal neurogenesis.

(a) The percentage of BrdU$^+$GFAP$^+$S100β$^-$ type 1 aNSCs among total BrdU$^+$ cells in the DG of GMF- and HMF-exposed mice 1-, 2-, 4-weeks after BrdU injection. (b) The percentage of BrdU$^+$Ki67$^+$DCX$^-$ transient amplifying cells among total BrdU$^+$ cells in the DG of GMF- and HMF-exposed mice 1-, 2-, 4-weeks after BrdU injection. (c) The percentage of BrdU$^+$Ki67$^+$DCX$^+$ neuroblasts among total BrdU$^+$ cells in the DG of GMF- and HMF-exposed mice 1-, 2-, 4-weeks after BrdU injection. (d) The percentage of BrdU$^+$DCX$^+$NeuN$^+$ immature neurons among total BrdU$^+$ cells in the DG of GMF- and HMF-exposed mice 1-, 2-, 4-weeks after BrdU injection. (e) The percentage of BrdU$^+$ NeuN$^+$ mature neurons among total BrdU$^+$ cells in the DG of GMF- and HMF-exposed mice 1-, 2-, 4-weeks after BrdU injection. (f) The percentage of BrdU$^+$ S100β$^+$ astrocytes among total BrdU$^+$ cells in the DG of GMF- and HMF-exposed mice 1-, 2-, 4-weeks after BrdU injection. GMF, n = 4 mice, HMF, n = 4 mice. All data are presented as mean ± SEM, and error bars were presented in SEM. Data were analyzed by two-way ANOVA, and the two-tailed unpaired *t*-test for two group comparisons at each time-point.

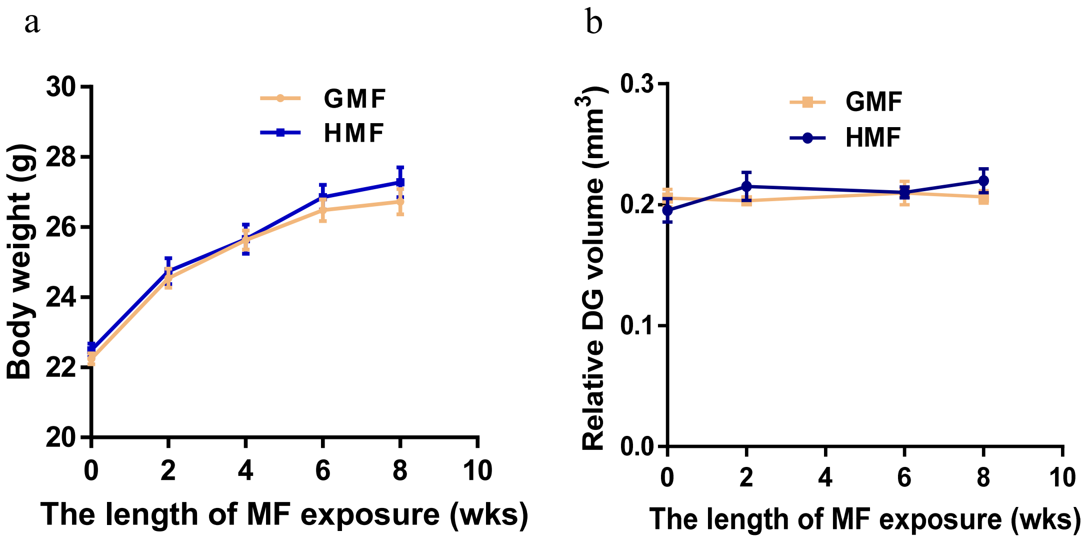

Supplementary Fig. 4. The body weight and DG volume of GMF- and HMF-exposed mice.

(a) The body weight of adult mice during GMF- or HMF-exposure (GMF, n = 10 mice, HMF, n = 10 mice, GMF versus HMF, two-way ANOVA, $F(1, 90) = 1.845$, $P = 0.1777$). (b) Quantification of the relative DG volume of GMF- and HMF-exposed mice (GMF, n = 4 mice, HMF, n = 4 mice, GMF versus HMF, two-way ANOVA, $F(1, 23) = 0.4326$, $P = 0.5172$). Data are presented as mean ± SEM, and error bars were presented in SEM.

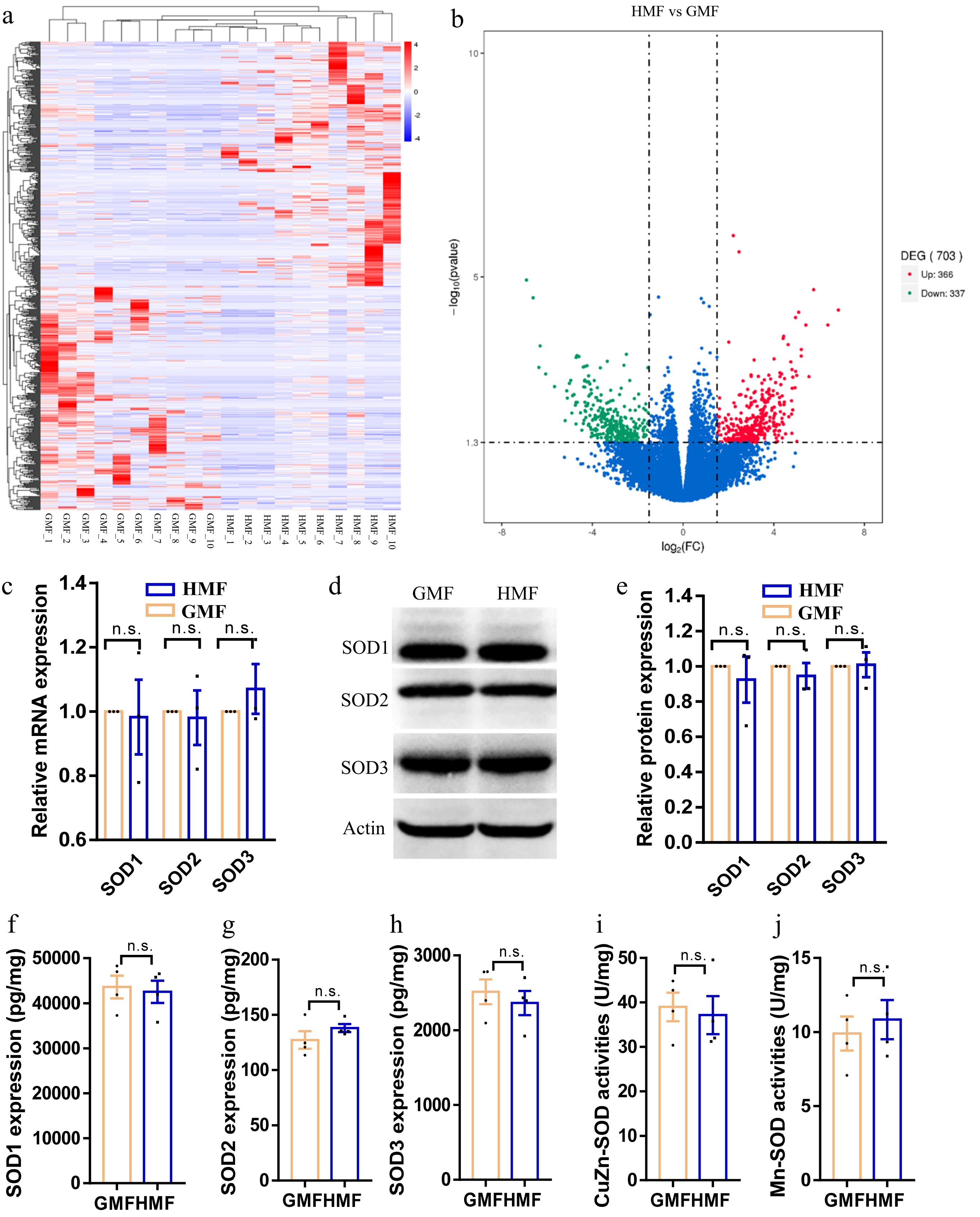

a
GMF_1
GMF_2
GMF_3
GMF_4
GMF_5
GMF_6
GMF_7
GMF_8
GMF_9
GMF_10
HMF_1
HMF_2
HMF_3
HMF_4
HMF_5
HMF_6
HMF_7
HMF_8
HMF_9
HMF_10
b
HMF vs GMF
DEG (703)
Up: 366
Down: 337
−log10(pvalue)
log2(FC)
c
HMF
GMF
n.s.
Relative mRNA expression
SOD1
SOD2
SOD3
d
GMF
HMF
SOD1
SOD2
SOD3
Actin
e
HMF
GMF
n.s.
Relative protein expression
SOD1
SOD2
SOD3
f
SOD1 expression (pg/mg)
n.s.
GMFHMF
g
SOD2 expression (pg/mg)
n.s.
GMFHMF
h
SOD3 expression (pg/mg)
n.s.
GMFHMF
i
CuZn-SOD activities (U/mg)
n.s.
GMFHMF
j
Mn-SOD activities (U/mg)
n.s.
GMFHMF

Supplementary Fig. 5. Long-term exposure to HMF downregulated the expression of mRNA transcripts associated with ROS generation in aNSCs.

(a) A heatmap of expression levels (log2RPKM; reads per kb of exon per million mapped reads) of differential expression genes in aNSCs isolated from GMF- and HMF-exposed Nestin-GFP mice and their replicates. (b) Volcano plot of differential expression genes. Red and green dots represent probe sets for transcripts expressed at significantly higher or lower levels in aNSCs isolated from GMF- and HMF-exposed mice, respectively. (c) RT-qPCR analysis of mRNA levels of SOD1, SOD2 and SOD3 in GMF- and HMF-cultured aNSCs. GMF, n = 3 mice, HMF, n = 3 mice. (d) Western blot of SOD1, SOD2 and SOD3 in GMF- and HMF-cultured aNSCs. (e) Quantification of western blot analysis on the protein levels of SOD1, and SOD3 in GMF- and HMF-cultured aNSCs. The samples derive from the same experiment (d) and that blots were processed in parallel. GMF, n = 3 mice, HMF, n = 3 mice. (f-h) Elisa analysis for the expression levels of SOD1 (f) , SOD2 (g) and SOD3 (h) protein expression levels in GMF- and HMF-cultured aNSCs. GMF, n = 3 mice, HMF, n = 3 mice. (i-j) The activities of CuZcn-SOD (i) and Mn-SOD (j) in GMF- and HMF-cultured aNSCs. GMF, n = 3 mice, HMF, n = 3 mice. All data are presented as mean ± SEM, and error bars were presented in SEM. *P*-values were determined from two-tailed unpaired *t*-test; n.s. = not significant.

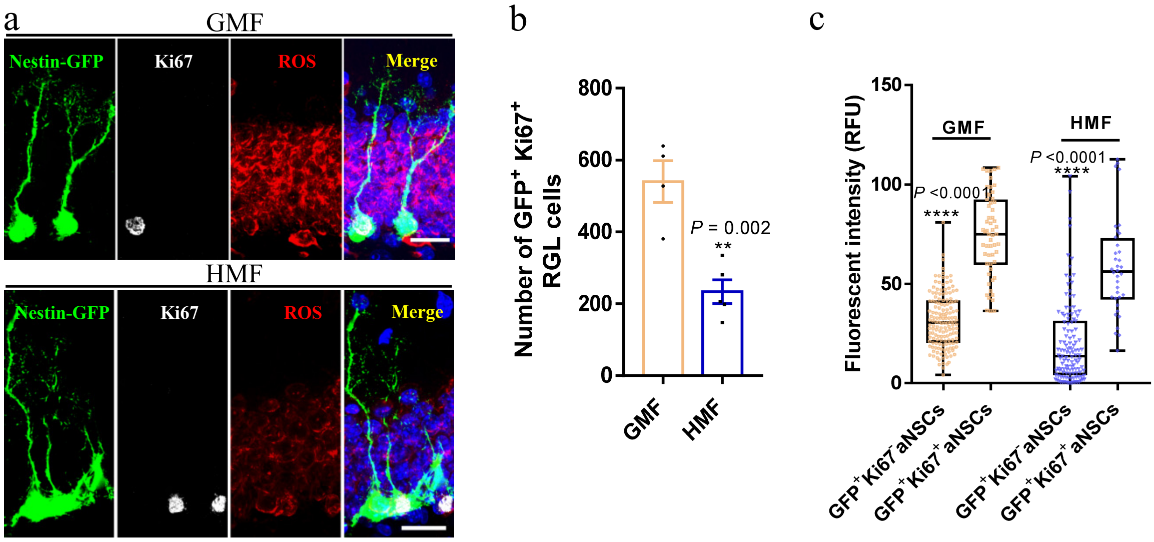

Supplementary Fig. 6. HMF exposure led to inhibit the activation of quiescent type 1 aNSCs

(a) Representative images of brain section co-labeled with GFP, Ki67 and ROS in the adult DG. Scale bar = 20 μm. (b) Quantification of numbers of $GFP^{+}Ki67^{+}$ cells of in the DG of Nestin-GFP mice after 8 weeks GMF and HMF exposure. GMF, n = 4 mice, HMF, n =4 mice. Data are presented as mean ± SEM, and error bars were presented in SEM. *P*-values were determined from two-tailed unpaired *t*-test. (c) Quantification of hydroethidine fluorescence insensitive of $GFP^{+}Ki67^{+}$ aNSCs and $GFP^{+}Ki67^{-}$ aNSCs in the DG of Nestin-GFP mice after 8 weeks GMF and HMF exposure (GMF, $GFP^{+}Ki67^{-}$ aNSCs = 154 cells from 4 mice, $GFP^{+}Ki67^{+}$ aNSCs = 57 cells from 4 mice; HMF, $GFP^{+}Ki67^{-}$ aNSCs = 125 cells from 4 mice, $GFP^{+}Ki67^{+}$ aNSCs = 37 cells from 4 mice). The data were presented in the whisker plot with defined elements, median (center line), upper and lower quartiles (bounds of box), and highest and lowest values (whiskers). *P*-values were determined from two-tailed unpaired *t*-test.

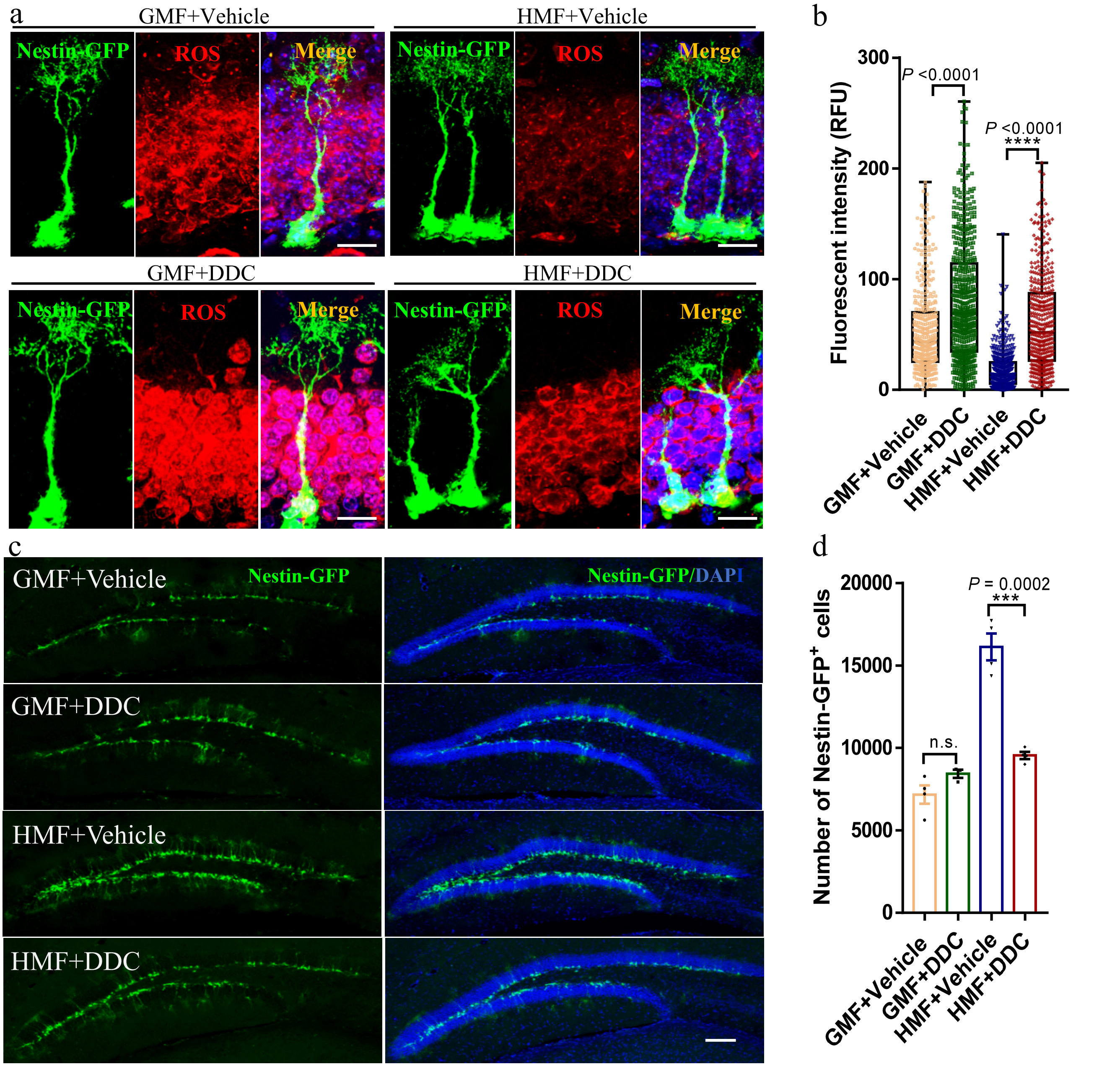

Supplementary Fig. 7. The effects of DDC treatment on ROS levels and GFP^{+} cells in GMF- and HMF-exposed Nestin-GFP mice.

(a) Hydroethidine fluorescence staining in the DG of GMF- and HMF-exposed Nestin-GFP mice with or without DDC treatment. Scale bar = 20 μm. (b) Quantification of hydroethidine fluorescence insensitive of GFP^{+} aNSCs in GFP^{+} cells of GMF- and HMF-exposed Nestin-GFP mice with or without DDC treatment, GMF, n = 410 cells from 4 mice , GMF+DDC, n = 557 cells from 4 mice, HMF, n = 525 cells from 4 mice, HMF+DDC, n = 442 cells from 4 mice. The data were presented in the whisker plot with defined elements, median (center line), upper and lower quartiles (bounds of box), and highest and lowest values (whiskers). *P*-values were determined from two-tailed unpaired *t*-test. (c) Representative images of GFP^{+} cells in the DG of GMF- and HMF-exposed Nestin-GFP mice with or without DDC treatment. Scale bar = 200 μm. (d) Quantification of the numbers of GFP^{+} cells in the DG of GMF- and HMF-exposed Nestin-GFP mice with or without DDC treatment, GMF+Vehicle, n = 4 mice, GMF+DDC, n = 3 mice, HMF+Vehicle, n = 4 mice, HMF+DDC, n = 4 mice. Data are presented as mean ± SEM, and error bars were presented in SEM. *P*-values were determined from two-tailed unpaired *t*-test; n.s. = not significant.

a

b

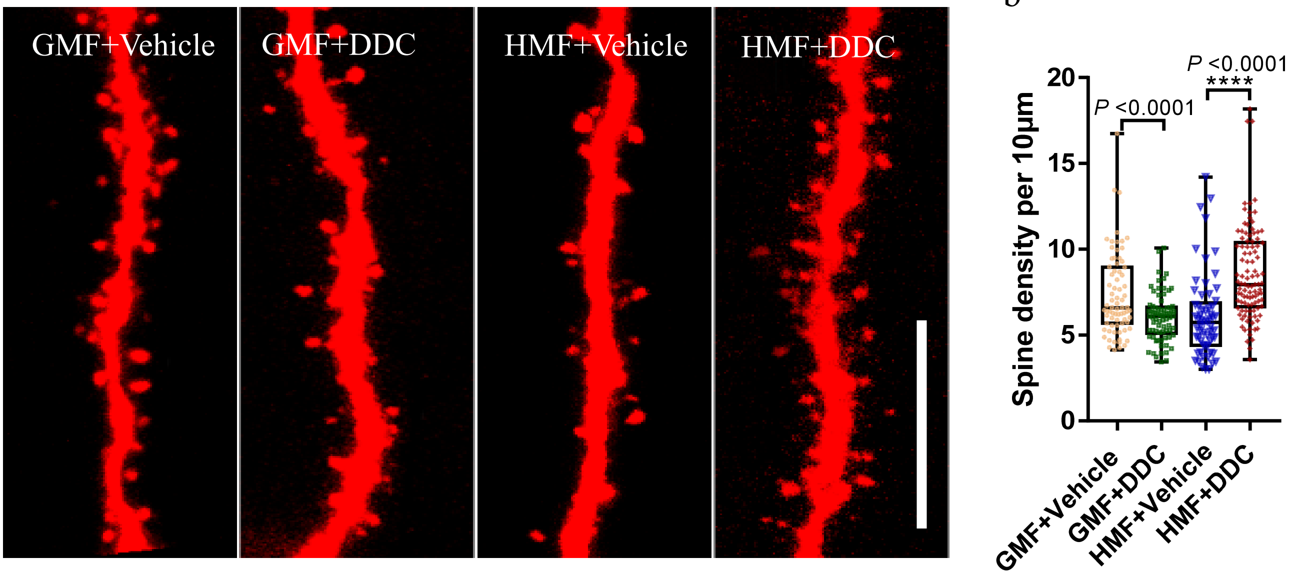

Supplementary Fig. 8. The effect of DDC treatment on spine development of newborn neurons in GMF- and HMF-exposed mice.

(a) Representative images of dendritic spines of newborn neurons in the DG in GMF- or HMF-exposed mice with or without DDC treatment. Scale bar = 10 μm. (b) Quantification of the numbers of dendritic of newborn neurons in the DG in GMF- or HMF-exposed mice with or without DDC, GMF+Vehicle, n = 75 from 4 mice, GMF+DDC, n = 74 from 4 mice, HMF+Vehicle, n = 61 from 4 mice, HMF+DDC, n = 102 from 4 mice. The data were presented in the whisker plot with defined elements, median (center line), upper and lower quartiles (bounds of box), and highest and lowest values (whiskers). *P*-values were determined from two-tailed unpaired *t*-test.

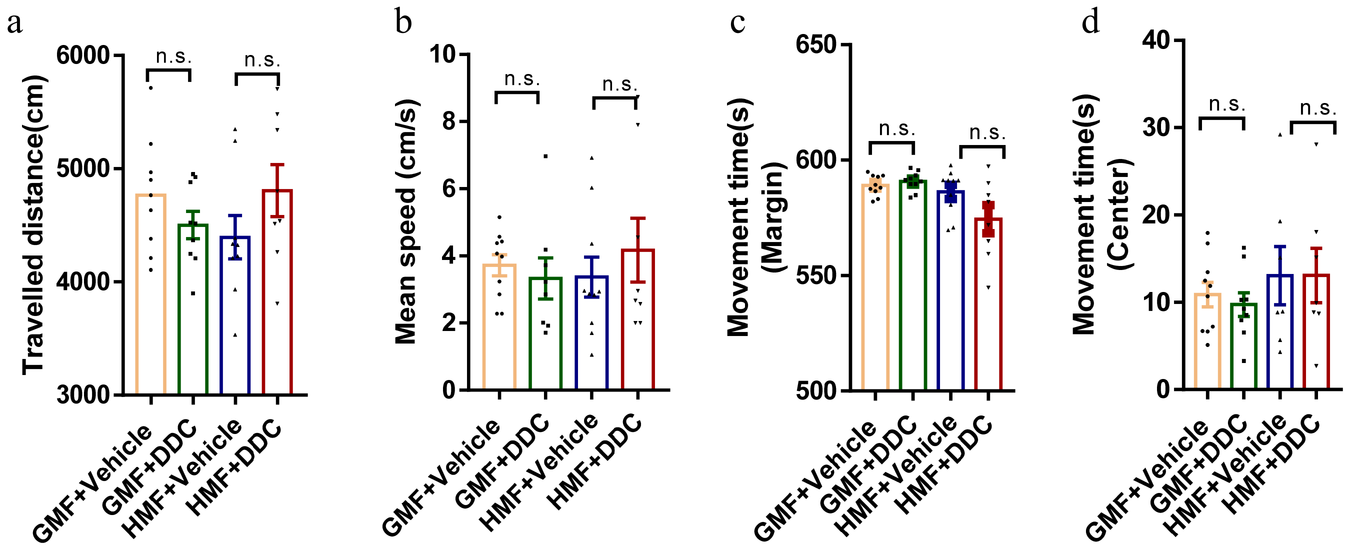

Supplementary Fig. 9. There were not deficits to open-filed test in HMF- and GMF-exposed mice with or without DDC treatment.

(a-b) There was no difference in the total distance traveled (a) or mean moving speed (b) between GMF- and HMF-exposed mice with or without DDC treatment in the open-field task. (c-d) The GMF- and HMF-exposed mice with or without DDC treatment did not exhibit reduced exploration in the margin (c) or center (d) of the arena, shown as the time spent in the margin or center of the arena during the open-field test. GMF+Vehicle, n = 9 mice, GMF+DDC, n = 9 mice, HMF+Vehicle, n = 9 mice, HMF+DDC, n = 8 mice. All data are presented as mean ± SEM, and error bars were presented in SEM. *P*-values were determined using two-tailed unpaired *t*-test; n.s. = not significant.

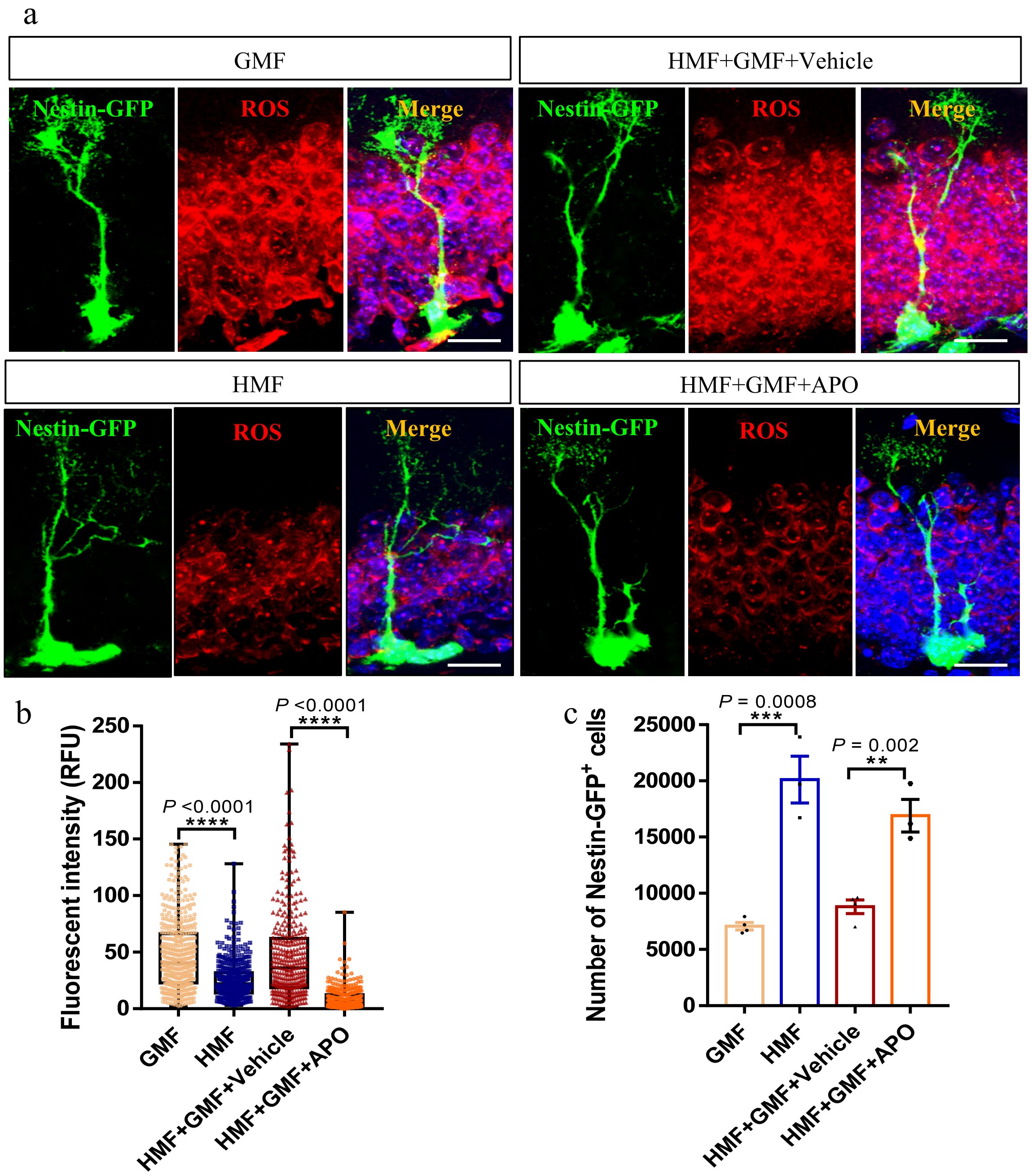

Supplementary Fig. 10. The promoting effects of return to GMF on ROS levels and GFP⁺ cell population in the DG of HMF-exposed Nestin-GFP mice were fully blocked by APO treatment.

(a) Hydroethidine fluorescence staining in the DG of HMF-exposed Nestin-GFP mice, followed by return to GMF with or without APO treatment. Scale bar = 20 μm. (b) Quantification of hydroethidine fluorescence insensitive of GFP^+ NPCs in aNSCs of HMF-exposed Nestin-GFP mice, followed by return to GMF with or without APO treatment, GMF, n = 481 cells from 4 mice, HMF, n = 463 cells from 3 mice, HMF+GMF+Vehicle, n = 342 cells from 4 mice, HMF+GMF+APO, n = 368 cells from 4 mice. The data were presented in the whisker plot with defined elements, median (center line), upper and lower quartiles (bounds of box), and highest and lowest values (whiskers). *P*-values were determined from two-tailed unpaired *t*-test. (c) Quantification of the numbers of GFP^+ cells in the DG of HMF-exposed Nestin-GFP mice, followed by return to GMF with or without APO treatment, GMF, n = 4 mice, HMF, n = 3 mice, HMF+GMF+Vehicle, n = 4 mice, HMF+GMF+APO, n = 4 mice. Data are presented as mean ± SEM, and error bars were presented in SEM. *P*-values were determined from two-tailed unpaired *t*-test.

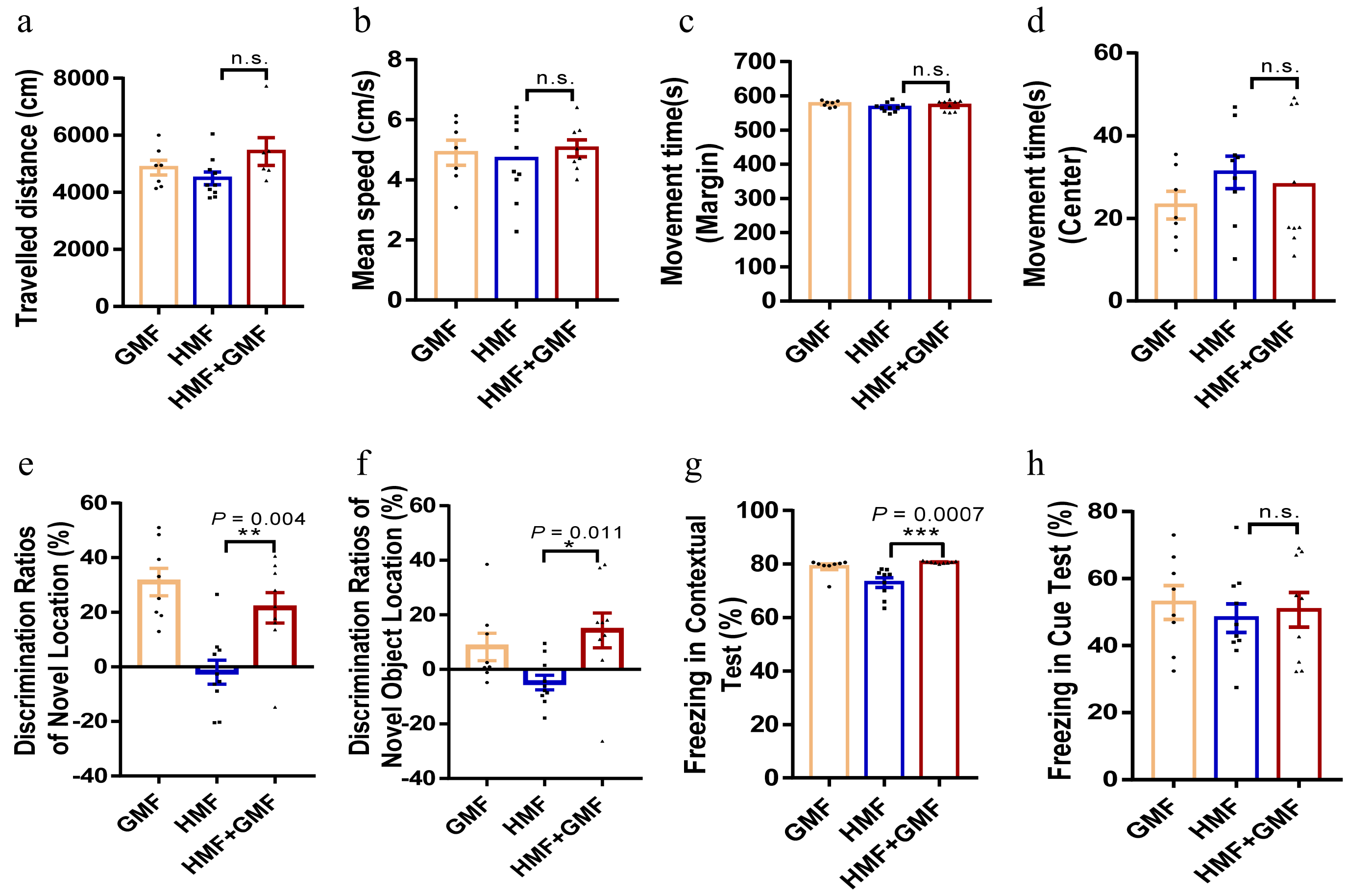

Supplementary Fig. 11. Return to GMF rescued defective hippocampus-dependent learning caused by HMF exposure.

(a-b) There was no difference in the total distance traveled (a) or mean moving speed (b) during the open-field task in HMF-exposed mice, followed by return to GMF. (c-d) The HMF-exposed mice, followed by return to GMF, did not exhibit reduced exploration in the margin (c) or center (d) of the arena, shown as the time spent in the margin or center of the arena during the open-field test. (e-f) Quantification of the ratio of exploration time on novel location (e) and novel object location (f) in HMF-exposed mice, followed by return to GMF. (g-h) The percentage analysis of freezing behavior during fear conditioning tests (g, contextual test,; h, cue test) in HMF-exposed mice, followed by return to GMF. GMF, n = 8 mice, HMF, n = 10 mice, HMF+GMF, n = 9 mice. All data are presented as mean ± SEM, and error bars were presented in SEM. *P*-values were determined using two-tailed unpaired *t*-test. test; n.s. = not significant.

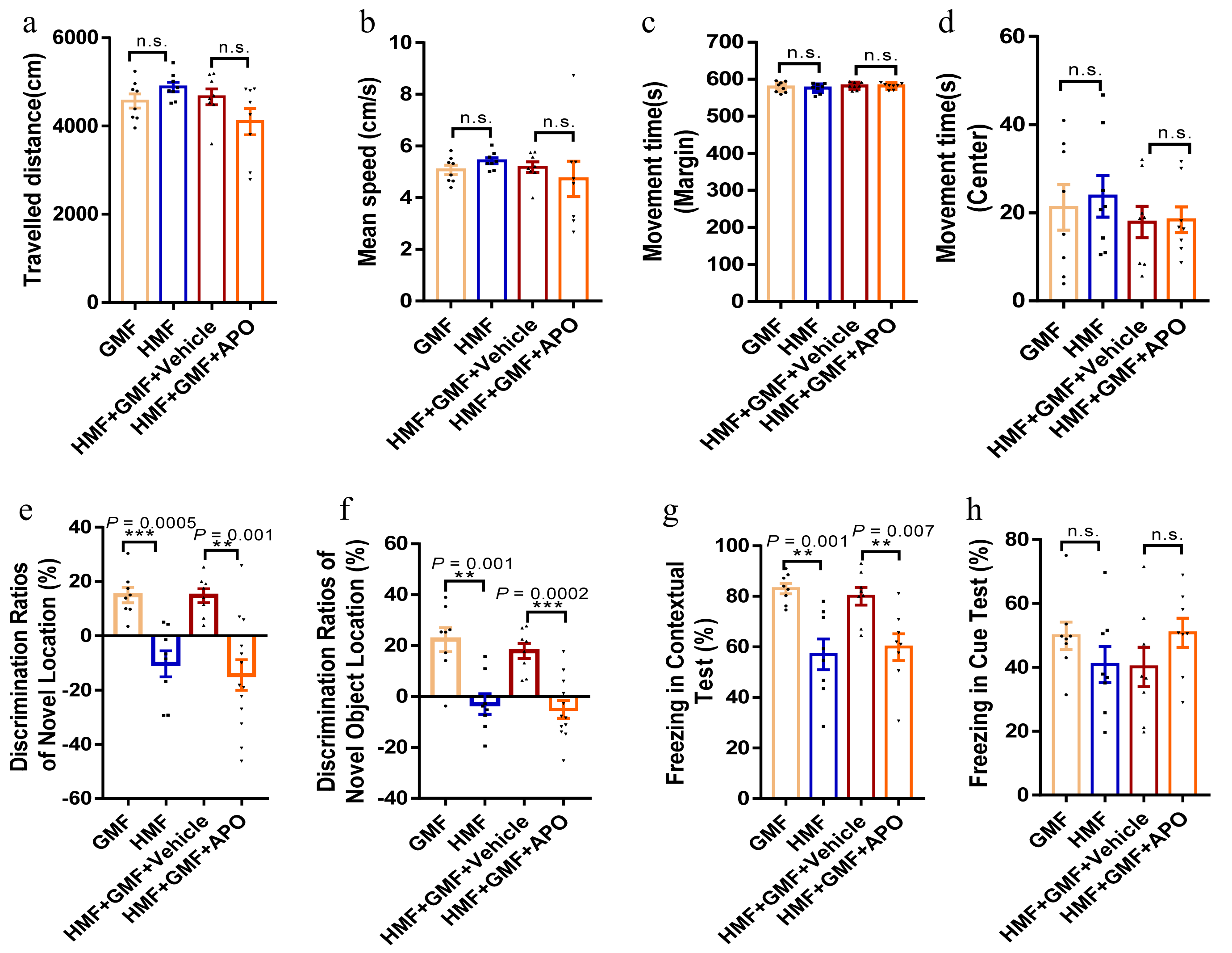

Supplementary Fig. 12. APO blocked the rescue effects of return to GMF on defective adult hippocampus-dependent learning of HMF-exposed mice.

(a-b) There was no difference in the total distance traveled (a) or mean moving speed (b) in HMF-exposed mice, followed by return to GMF with or without APO treatment in the open-field task. (c and d) The GMF- and HMF-exposed mice, followed by return to GMF with or without APO treatment, did not exhibit reduced exploration in the margin (c) or center (d) of the arena, shown as the time spent in the margin or center of the arena during the open-field test. (e-f) Quantification of the ratio of exploration time on novel location (e) and novel objective object (f) in HMF-exposed mice, followed by return to GMF with or without APO treatment. (g-h) The percentage analysis of freezing behavior during fear conditioning tests (g, the contextual test; h, cue test) in HMF-exposed mice followed by return to GMF with or without APO treatment. GMF, n = 8 mice, HMF, n = 8 mice, HMF+GMF+Vehicle, n = 8 mice, HMF+GMF+APO, n = 8 mice. All data are presented as mean ± SEM, and error bars were presented in SEM. *P*-values were determined using two-tailed unpaired *t*-test. n.s. = not significant.

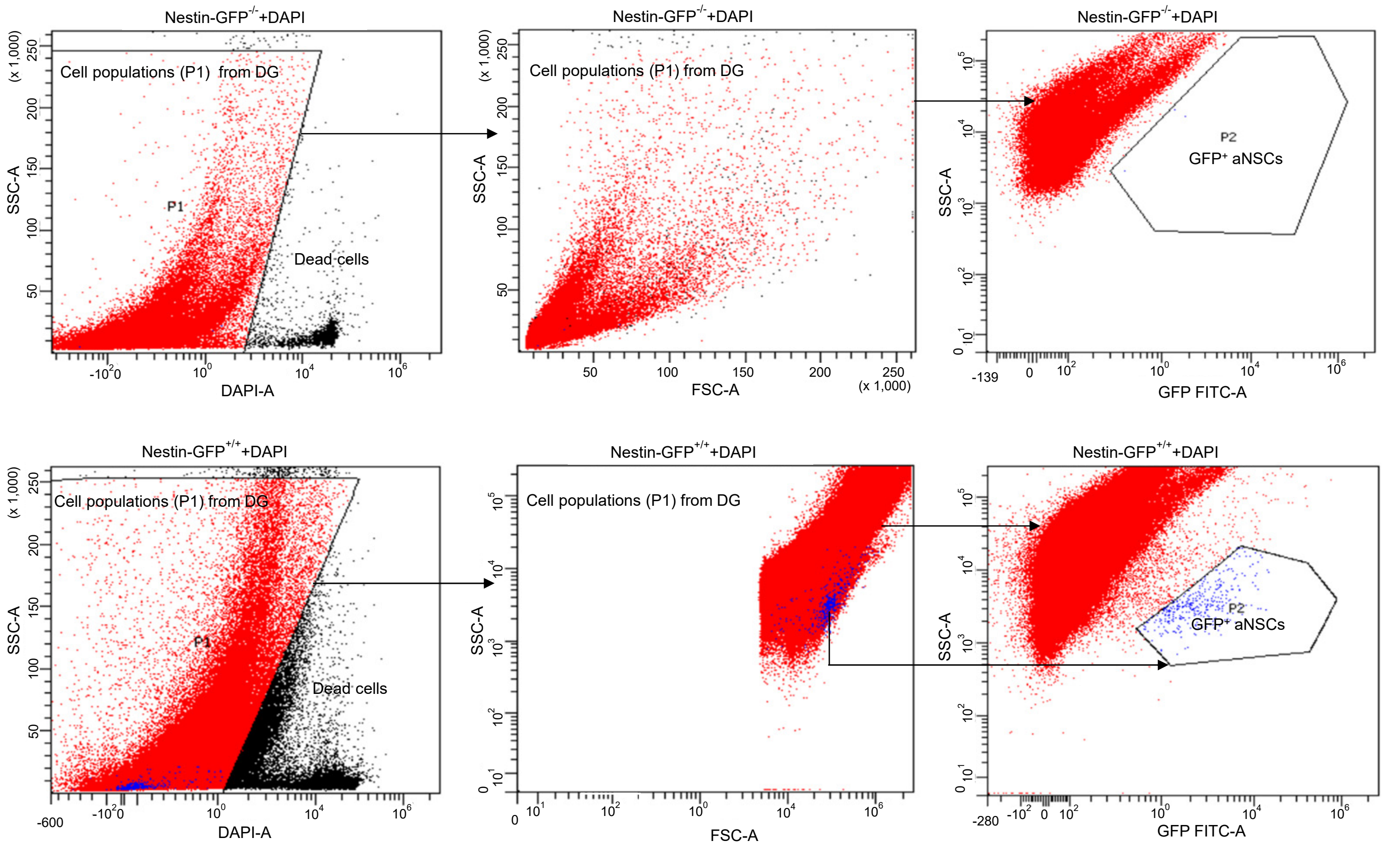

Supplementary Fig. 13. Gating strategy for detection of GFP+ aNSCs (P2) within cell populations (P1) from DG of mice without Nestin-GFP transgene(upper panel) or with Nestin-GFP transgene (bottom panel).

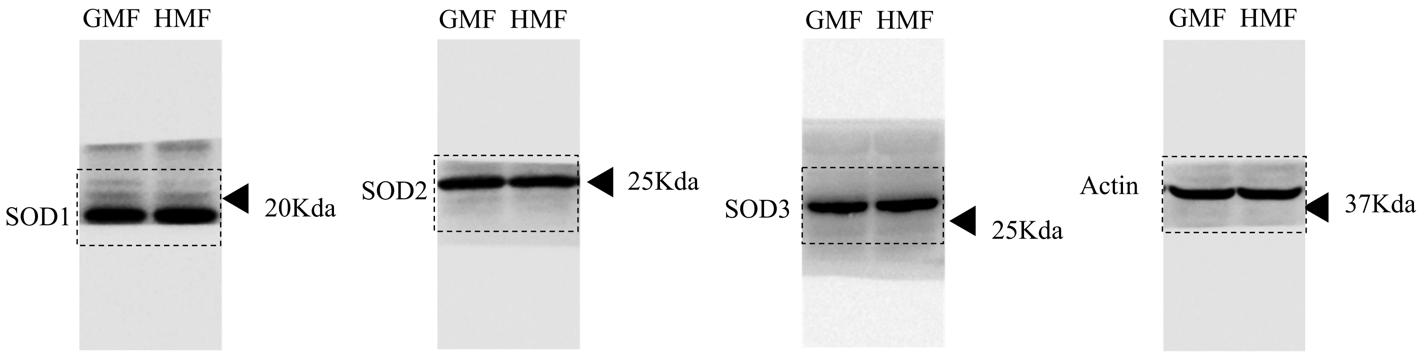

Supplementary Fig. 14. The full blot used for supplementary figure 5d.

Western blot of SOD1, SOD2 , SOD3 and Actin in GMF- and HMF-cultured aNSCs.

Supplementary table 1

Number of mice and exposure time in each experiment

Experiment name	Exposure time	Number of mice
aNSCs proliferation	0-,2-, 6-, 8-weeks	4 mice per group in HMF/GMF
aNSCs differentiation	9-,11-,12-weeks	4 mice per group in HMF/GMF
dendritic development of neurons	4-weeks without HMF pre-exposure	4 mice per group in HMF/GMF
dendritic development of neurons	4-weeks with 8-weeks HMF pre-exposure	4 mice per group in HMF/GMF
RNA-sequencing	8-weeks	10 mice per group in HMF/GMF
RT-qPCR analysis	8-weeks	3 mice per group in HMF/GMF
fluorescence insensitive	8-weeks	4 mice per group in HMF/GMF
Nestin-GFP + cells	8-weeks	10 mice per group in HMF/GMF
aNSCs proliferation with DDC treatment	4+4 weeks	4 mice per group in HMF/GMF/HMF+DDC/GMF+DDC
aNSCs differentiation with DDC treatment	4+4 weeks	4 mice per group in HMF/GMF/HMF+DDC/GMF+DDC
dendritic development of neurons with DDC treatment	4+4 weeks	4 mice per group in HMF/GMF/HMF+DDC/GMF+DDC
Behavioral tests with DDC treatment	4+4 weeks	9 mice per group in HMF/GMF/ GMF+DDC; 8 mice in HMF+DDC
fluorescence insensitive and Nestin-GFP + cells with DDC treatment	4+4 weeks	4 mice per group in HMF/GMF/HMF+DDC/GMF+DDC
aNSCs proliferation with GMF return	8 +2-, 4-, 6-weeks	4 mice per group in HMF/GMF/HMF+GMF
aNSCs differentiation with GMF return treatment	8 + 4-weeks	4 mice per group in HMF/GMF/HMF+GMF
dendritic development of neurons with GMF return	8 + 4-weeks	4 mice per group in HMF/GMF/HMF+GMF
dendritic development of neurons with GMF return	8 + 8-weeks	4 mice per group in HMF/GMF/HMF+GMF
Behavioral tests with GMF return	8 + 4-weeks	8 mice GMF; 10 mice in HMF; 9 mice in HMF+GMF
aNSCs proliferation with APO treatment	8 + 4-weeks	4 mice per group in HMF/GMF/HMF+APO/GMF+APO
aNSCs differentiation with APO treatment	8 + 4-weeks	4 mice per group in HMF/GMF/HMF+APO/GMF+APO
dendritic development of neurons with APO treatment	8 + 4-weeks	4 mice per group in HMF/GMF/HMF+APO/GMF+APO
dendritic development of neurons with APO treatment	8 + 8-weeks	4 mice per group in HMF/GMF/HMF+APO/GMF+APO
fluorescence insensitive and Nestin-GFP + cells with APO treatment	8 + 4-weeks	3 mice in HMF; 4 mice per group in GMF/HMF+APO/GMF+APO
Behavioral tests with APO treatment	8 + 4-weeks	8 mice per group in HMF/GMF/HMF+DDC/GMF+DDC

Supplementary table 2

AC magnetic field conditions

	Bt PSD$^{1/2}$ (nT/√Hz)	Dominate Frequency (Hz)
HMF in Helmholtz cage	2.37	50
GMF in control Helmholtz cage	6.80	50
HMF in magnetic shielding chamber	1.47	50
GMF in the cell incubator	4.65	50

Supplementary table 3

The primers used for RT-PCR

Names	Sense sequences	Anti-sense sequences
SOD1	ACTCTAAGAAACATGGTGGCCC	CTTCATTTCCACCTTTGCCCAA
Tpo	CTTTCTTTAAGCTGCCCGAGTC	CTGTGATGGGCCGGTACTTATT
Akr1c13	TGGATTTCTGTGACACATGGGA	GCTTACGCTGGTTCAAATAGAGA
Art5	CTTGTTTTGGGGCTCCTATCCA	TTAAAGTGGCTGCAGGTCTGAT
Gm4952	GTGGCCTGATTTTAATACAGTGGT	TGCACTGTGTTCTCTTGACCTT
Gzmc	AGGCAATGAGATCAGTCCACAT	ATAGTCTGGATGGGGAATGGCT
Cyp4f37	TGAGGATTGTTGACCCTGCATT	TGCATGATGTTCACACTCTGGT
Rgn	CGATTCAATGATGGGAAGGTGG	AGGTCATAGTCAAAGGCATCCA
Hba-x	TTCAAGATCATGACCGCCGTAG	ACATGAACTTGTCCCAGGCTT
Cyp2d11	ATCAATCCCAACACCATGCTGA	AAGGCATCAGTCAAATTTCGGG
Mboat4	TGTGCTGGTCTGTAATGGTGAA	GTAAGAACAGCACAGTTGGCAG
Nipsnap3a	TTGGACTGTAGGATTTGGAGGC	AAATGCTTCACCCCATAGAGCT
Atp1a4	TACTAACTCCTCCTCCAACCACT	TGGTAATATGAGAAGCAGCCGG
Cyp7a1	TGAAGCAATGAAAGCAGCCTCT	AGGTGTAGAGTGAAGTCCTCCT
GAPDH	AATGGGAAGCTTGTCATCAACG	GAAGACACCAGTAGACTCCACGACATA
