# Supplementary material for: Long-term exposure to a hypomagnetic field attenuates adult hippocampal neurogenesis and cognition
Source: Nat Commun. 2021 Feb 19;12:1174. doi: 10.1038/s41467-021-21468-x (PMC7896063; doi:10.1038/s41467-021-21468-x)
Supplement: Supplementary file 2 — Reporting Summary [file 41467_2021_21468_MOESM2_ESM.pdf]

nature research

Corresponding author(s): Weixiang Guo
Lanxiang Tian

Last updated by author(s): Jan 13, 2021

Reporting Summary

Nature Research wishes to improve the reproducibility of the work that we publish. This form provides structure for consistency and transparency in reporting. For further information on Nature Research policies, see our Editorial Policies and the Editorial Policy Checklist.

Statistics

For all statistical analyses, confirm that the following items are present in the figure legend, table legend, main text, or Methods section.

n/a	Confirmed	
☐	☒	The exact sample size (*n*) for each experimental group/condition, given as a discrete number and unit of measurement
☐	☒	A statement on whether measurements were taken from distinct samples or whether the same sample was measured repeatedly
☐	☒	The statistical test(s) used AND whether they are one- or two-sided<br>*Only common tests should be described solely by name; describe more complex techniques in the Methods section.*
☒	☐	A description of all covariates tested
☒	☐	A description of any assumptions or corrections, such as tests of normality and adjustment for multiple comparisons
☐	☒	A full description of the statistical parameters including central tendency (e.g. means) or other basic estimates (e.g. regression coefficient) AND variation (e.g. standard deviation) or associated estimates of uncertainty (e.g. confidence intervals)
☐	☒	For null hypothesis testing, the test statistic (e.g. *F*, *t*, *r*) with confidence intervals, effect sizes, degrees of freedom and *P* value noted<br>*Give P values as exact values whenever suitable.*
☒	☐	For Bayesian analysis, information on the choice of priors and Markov chain Monte Carlo settings
☒	☐	For hierarchical and complex designs, identification of the appropriate level for tests and full reporting of outcomes
☒	☐	Estimates of effect sizes (e.g. Cohen's *d*, Pearson's *r*), indicating how they were calculated

Our web collection on statistics for biologists contains articles on many of the points above.

Software and code

Policy information about availability of computer code

Data collection	Confocal imaging (Olympus,System FV 1000; VS100),NIS-Elements, BR. 3.00 software, ImageJ for imaging analysis (RRID: SCR_003070), Panlab SMART 3.0 Software, Corel VideoStudio Pro X5, Packwin software (V2.0.05)
Data analysis	Data were analyzed with Graphpad Version 6.01, Image J-win64, Olympus FV10-ASW 2.0 Viewer, Adobe Illustrator CS6 (version 16), Adobe Photoshop CC 2015, Microsoft office 2011, IBM SPSS_22.0 software.

For manuscripts utilizing custom algorithms or software that are central to the research but not yet described in published literature, software must be made available to editors and reviewers. We strongly encourage code deposition in a community repository (e.g. GitHub). See the Nature Research guidelines for submitting code & software for further information.

Data

Policy information about availability of data

All manuscripts must include a data availability statement. This statement should provide the following information, where applicable:

- Accession codes, unique identifiers, or web links for publicly available datasets
- A list of figures that have associated raw data
- A description of any restrictions on data availability

Source data are provided with this paper. All data are contained in the main text and the supplementary materials, and are provided as a Source Data file Figshare (https://figshare.com/s/dc08672eab2dcd45f4dd). All raw sequenced data are stored in the Sequence Read Archive (SRA, PRJNA643878) (https://www.ncbi.nlm.nih.gov/Traces/study/?acc=PRJNA643878).

Field-specific reporting

Please select the one below that is the best fit for your research. If you are not sure, read the appropriate sections before making your selection.

- [x] Life sciences
- [ ] Behavioural & social sciences
- [ ] Ecological, evolutionary & environmental sciences

For a reference copy of the document with all sections, see nature.com/documents/nr-reporting-summary-flat.pdf

Life sciences study design

All studies must disclose on these points even when the disclosure is negative.

Sample size	Sample size was used as dictated by literature on similar studies. The statistical significance of the result we obtained justifies the sample size we used. PMID:30497772, PMID:30531936, PMID: 12748381, PMID: 20386739, PMID: 20452318, PMID: 30497772
Data exclusions	No data were excluded from the analyses.
Replication	The experiment for each experiment was successfully repeated for at least three times.
Randomization	cells analyzed were randomly selected from in vivo and in vitro samples.
Blinding	All experiments and data analyses were conducted in a blinded manner, including the immunohistochemistry, dendritic morphology analyses, ELISA and behavioral analyses. Quantifications were performed by experimenters who were blind to the identity of the sample.

Reporting for specific materials, systems and methods

We require information from authors about some types of materials, experimental systems and methods used in many studies. Here, indicate whether each material, system or method listed is relevant to your study. If you are not sure if a list item applies to your research, read the appropriate section before selecting a response.

Materials & experimental systems

n/a	Involved in the study	
[ ]	[x]	Antibodies
[x]	[ ]	Eukaryotic cell lines
[x]	[ ]	Palaeontology and archaeology
[ ]	[x]	Animals and other organisms
[x]	[ ]	Human research participants
[x]	[ ]	Clinical data
[x]	[ ]	Dual use research of concern

Methods

n/a	Involved in the study	
[x]	[ ]	ChIP-seq
[ ]	[x]	Flow cytometry
[x]	[ ]	MRI-based neuroimaging

Antibodies

Antibodies used	The primary antibodies used:rat anti-BrdU (1:1000, Abcam, ab6326), goat anti-GFAP (1:1000, Abcam, ab53554), mouse anti-SOX2 (1:1000, Abcam, ab79351), rabbit anti-Ki67 (1:500, Invitrogen, MA5-14520), mouse anti-NeuN (1:500, Millipore, MAB377), rabbit anti-S100β (1:1000, Dako, Z0334), goat anti-DCX (1:100, Santa Cruz Biotechnology, SC-8066), rabbit anti-cleaved caspase-3(Asp175) (1:500, Cell Signaling Technology, #96644), rabbit anti-SOD1 (1:500, Abcam, ab13498), rabbit anti-β-Actin (1:1000, Abcam, ab8227).<br>Fluorescent secondary antibodies for IHC used by 1:500 dilution:donkey anti-rat Alexa Fluor 488 (Invitrogen, A21208), donkey anti-goat Alexa Fluor 568 (Invitrogen, A11057), donkey anti-mouse Alexa Fluor 647 (Invitrogen, A31571), donkey anti-rabbit Alexa Fluor 488 (Invitrogen, A21206), donkey anti-rabbit Alexa Fluor 647 (Invitrogen, A31573), donkey anti-mouse Alexa Fluor 568 (Invitrogen, A10037), donkey anti-goat 647 (Invitrogen, A21447), and donkey anti-rabbit Alexa Fluor 568 (Invitrogen, A10042).<br>Secondary antibodies for WB used by 1:5000 dilution: HRP-conjugated Goat anti-Rabbit (Abcam, ab6721).
Validation	1.rat anti-BrdU (Abcam, ab6326),rat, IHC, validation on manufacturer's website:https://www.abcam.cn/brdu-antibody-bu175-icr1-ab6326.html<br>2.goat anti-GFAP (Abcam, ab53554), goat, IHC, validation on manufacturer's website:https://www.abcam.cn/gfap-antibody-ab53554.html<br>3.mouse anti-SOX2 (Abcam, ab79351), mouse, IHC, validation on manufacturer's website:https://www.abcam.cn/sox2-antibody-9-9-3-ab79351.html<br>4.rabbit anti-Ki67 (Invitrogen, MA5-14520), rabbit, IHC, validation on manufacturer's website:https://www.thermofisher.com/antibody/product/Ki-67-Antibody-clone-SP6-Monoclonal/MA5-14520<br>5.mouse anti-NeuN (Millipore, MAB377), mouse, IHC, validation on manufacturer's website:https://www.merckmillipore.com/CN/zh/product/Anti-NeuN-Antibody-clone-A60,MM_NF-MAB377<br>6.rabbit anti-S100β (Dako, Z0334), validation on publication: PMID: 28100736.<br>7. goat anti-DCX (Santa Cruz Biotechnology, SC-8066), validation on publication: PMID: 22446882

8.rabbit anti-cleaved caspase-3 (Cell Signaling Technology, #96644), mouse, IHC, validation on manufacturer's website: https://www.cellsignal.com/products/primary-antibodies/cleaved-caspase-3-asp175-antibody/9664.
9.rabbit anti-SOD1 (1:500, Abcam, ab13498), rabbit, WB, validation on manufacturer's website: https://www.abcam.cn/superoxide-dismutase-1-antibody-ab13498.html
10.rabbit anti-β-Actin (1:1000, Abcam, ab8227),rabbit, WB, validation on manufacturer's website:https://www.abcam.cn/beta-actin-antibody-ab8227.html

Animals and other organisms

Policy information about studies involving animals; ARRIVE guidelines recommended for reporting animal research

Laboratory animals	Adult male mice expressing GFP in nestin-positive cells and wild-type C57BL/6J mice(8 weeks, 26-30g) were used.Mice were housed in the animal facility with a constant temperature and relative humidity (22±1 °C; 65±1%)
Wild animals	no wild animals were used
Field-collected samples	no field-collected samples were used
Ethics oversight	Institute of Genetics and Developmental Biology Animal Care and Use committees

Note that full information on the approval of the study protocol must also be provided in the manuscript.

Flow Cytometry

Plots

Confirm that:

- [x] The axis labels state the marker and fluorochrome used (e.g. CD4-FITC).
- [x] The axis scales are clearly visible. Include numbers along axes only for bottom left plot of group (a 'group' is an analysis of identical markers).
- [x] All plots are contour plots with outliers or pseudocolor plots.
- [x] A numerical value for number of cells or percentage (with statistics) is provided.

Methodology

Sample preparation	DG tissue were isolated from 16 weeks old Nestin-GFP mice and their WT littermates.
Instrument	Becton Dickinson FACS Aria II
Software	StereoInvestigator software (MicroBrightField)
Cell population abundance	All cell populations were isolated into single cells using a Becton Dickinson FACS Aria II contained in a Biosafety Carbinet using 20 psi pressure and 100-μm nozzle aperture. 10,000 total Nestin-GFP+ alive Cells were collected directly in Trizol.
Gating strategy	Gates were set manually by using control samples (same types of cells isolated from mice without Nestin-GFP transgene).

- [x] Tick this box to confirm that a figure exemplifying the gating strategy is provided in the Supplementary Information.
